# Hepatic stem cell *Numb* gene is a potential target of Huang Qi Decoction against cholestatic liver fibrosis

**DOI:** 10.1038/s41598-020-74324-1

**Published:** 2020-10-15

**Authors:** Wen Xu, Yan-nan Xu, Xu Zhang, Ying Xu, Xun Jian, Jia-mei Chen, Gao-feng Chen, Hua Zhang, Ping Liu, Yong-ping Mu

**Affiliations:** 1 Shuguang Hospital Affiliated to Shanghai University of Traditional Chinese Medicine (TCM); Institute of Liver Diseases, Key Laboratory of Liver and Kidney Diseases, Shanghai University of TCM, 528, Zhangheng Road, Pudong district, Shanghai, 201203 People’s Republic of China; 2 Shanghai Key Laboratory of TCM, Shanghai, People’s Republic of China; 3 E-Institute of Shanghai Municipal Education Commission, Shanghai University of TCM, Shanghai, People’s Republic of China

**Keywords:** Molecular biology, Stem cells, Gastroenterology

## Abstract

Numb is a negative regulator of Notch signal pathway. Previous study has demonstrated that Notch signal pathway activation is required for hepatic progenitor cell (HPC) differentiating into cholangiocytes in cholestatic liver fibrosis (CLF), and Huang Qi Decoction (HQD) could prevent CLF through inhibition of the Notch signal pathway. However, the role of Numb in HQD against CLF is yet unclear. Thus, CLF rats transplanted into rat bone marrow-derived mesenchymal stem cells with knocked down *Numb* gene (BMSC^*Numb*-KD^) were treated with HQD. Simultaneously, *Numb* gene knockdown was also performed in WB-F344 cell line and then treated with refined HQD in vitro. In vivo study revealed that liver fibrosis was inhibited by HQD plus BMSC^*Numb*-KD^ treatment, while Hyp content in liver tissue, the gene and protein expression of α-SMA, gene expression of Col I, TNF-α, and TGF-β1 were increased compared to that in HQD group. Furthermore, Notch signal pathway was inhibited by HQD plus BMSC^*Numb*-KD^, while the protein expression of Numb was decreased and RBP-Jκ and Hes1 was increased compared to that in HQD group. In vitro, HQD reduced the differentiation of WB-F344 cells into cholangiocyte phenotype, while this effect was attenuated after *Numb*-knockdown. This study highlights that the absence of hepatic stem cell *Numb* gene decreases effect of HQD against CLF, which give rise the conclusion that *Numb* might be a potential target for HQD against CLF.

## Introduction

Human cholestatic liver disease is characterized by the progressive destruction of biliary epithelial cells (BECs), followed by fibrosis, cirrhosis and liver failure^1,2^. Thus, the inhibition of abnormal BEC activation and proliferation may partially or completely reverse cholestatic liver fibrosis (CLF)^3^. In patients with advanced primary biliary cholangitis (PBC), ursodeoxycholic acid (UDCA) is effective to improve patient survival rates^4^, while cannot reverse PBC related liver fibrosis^5^. Therefore, it is necessary to explore the effective strategy for CLF.

Notch signal pathway is a highly conserved cell communication system that regulates a wide variety of cell types and cellular processes, such as apoptosis, proliferation, cell fate regulation, and asymmetric division^6^. There are four Notch receptors (Notch1–4) and five typical ligands (Delta-like (DLL)-1/-3/-4, and Jagged (Jag)-1/-2) in mammals. The interaction of Notch ligands with their receptors promotes a γ-secretase dependent cleavage of the Notch receptor and release of the Notch intracellular domain (NICD), which results in activation of the pathway^7,8^. NICD translocates to the nucleus and induces target genes like Hairy enhancer of split (Hes1)^9^. Numb, an important cell fate determinant, which is located in the endosome of eukaryotic cells and negative regulator of the Notch signal pathway^10,11^. Our previous study showed that hepatic progenitor cells (HPCs) are the main source of BECs in CLF rat induced by bile duct ligation (BDL), and Notch signaling activation was found to be required for this pathological process^12^, suggesting that inhibition of Notch signal pathway might be of profound significance for treatment of CLF.

Huang Qi Decoction (HQD), a classic Traditional Chinese Medicine formula, with rather good therapeutic effect on chronic liver diseases, is of two herb ingredients: Radix Astragali [*Astragalusmembranceus Fisch. (Bge.),* root, Huangqi] and Radix Glycyrrhizae (*Glycyrrhizauralensis Fisch.,* root and rhizome, Gancao). Cheng et al.^13^ reported that HQD is of good efficacy on cirrhosis due to chronic hepatitis B. Our previous study showed that HQD can inhibit the progression of CLF through inhibiting the Notch signal pathway and promoting the expressions of Numb mRNA and protein in hepatic progenitor cells^14^. Up to now, the role of Numb in the anti-CLF effect of HQD remains unclear. In this study, CLF rat transplanted into rat bone marrow-derived mesenchymal stem cells with *Numb* knockdown (BMSC^*Numb*-KD^) were found to promote CLF progression and reduced the effect of HQD against CLF.

## Materials and methods

### Materials

HQD, which contains 30 g of Radix Astragali [*Astragalusmembranceus Fisch.* (*Bge*.), root, Huangqi] and 5 g of Radix Glycyrrhizae (*Glycyrrhizauralensis Fisch*., root and rhizome, Gancao), was obtained from the Shanghai Huayu Herbs Co. Ltd. (Shanghai, China) and was accredited and prepared by pharmacognosist of Shuguang Hospital affiliated to Shanghai University of TCM and was maintained at − 20 °C. HQD fingerprint was obtained by ultra-high performance liquid chromatography Q-Orbitraphigh resolution mass spectrometry (UHPLC-Q-Orbitrap-HRMS), with the results shown in Supplementary Fig. 1.

**Figure 1:**
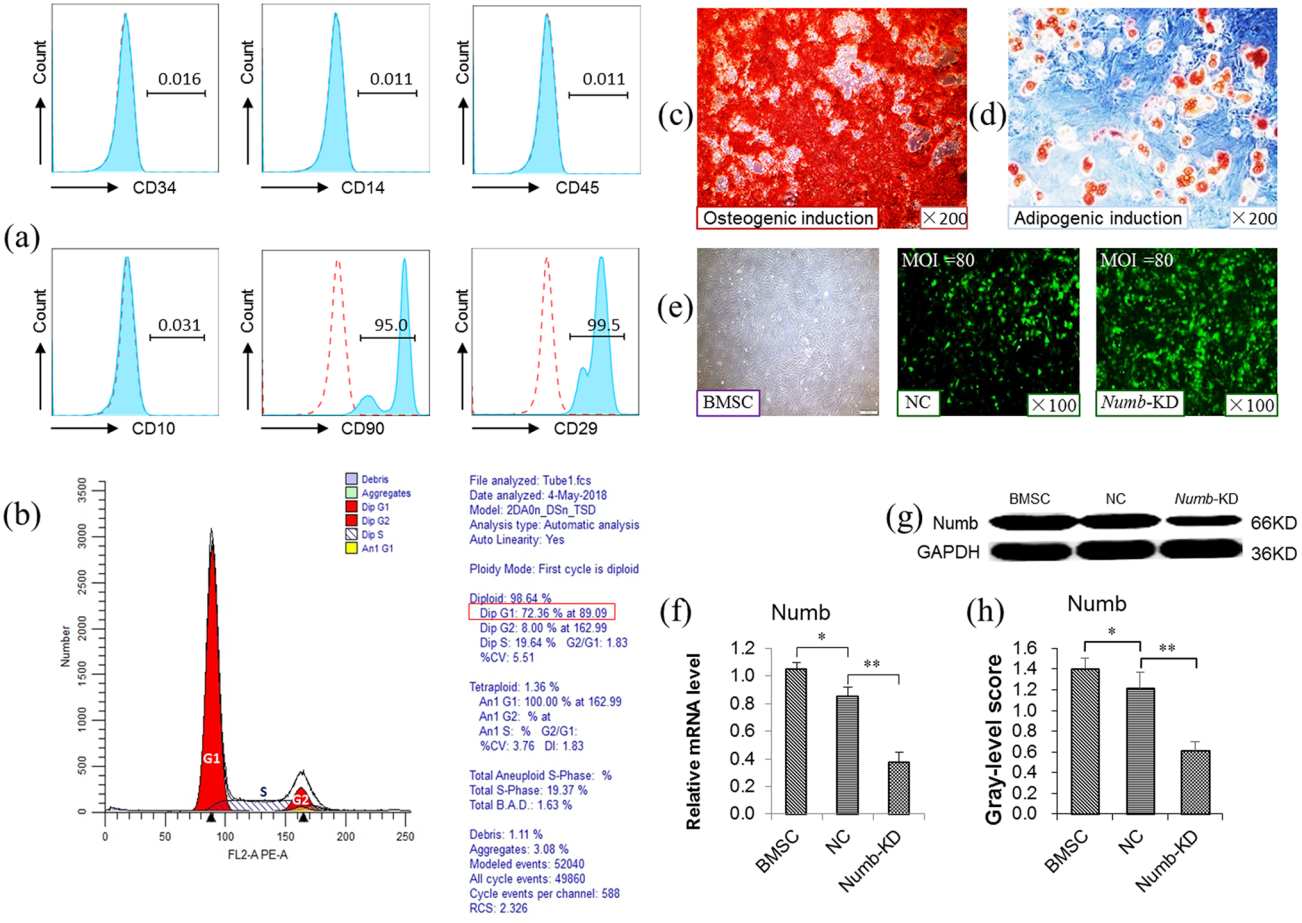
Lentivirus-mediated knockdown of the BMSC *Numb* gene. (**a**) Flow cytometry analysis of CD10, CD14, CD29, CD34, CD45, and CD90. (**b**) Flow cytometry analysis of BMSC proliferative capacity. (**c**) Osteogenic induction (×200). (**d**) Adipogenic induction (×200). (**e**) Lentivirus transfected BMSCs. (**f**) The relative Numb mRNA level determined by qRT-PCR, mRNA level were normalized by GAPDH (*n* = 3 per group). (**g**) Immunobloting for Numb, and (**h**) The gray-level score indicates the histogram of immunoblotting for Numb (*n* = 3 per group). *P < 0.05, **P < 0.01. BMSC, bone marrow-derived mesenchymal stem cell group; NC, empty virusgroup; *Numb*-KD, *Numb* knockdown group.

DAPT (a prototypical gamma-secretase inhibitor, and inhibiting gamma-secretase can prevent Notch receptor cleavage and thereby block Notch signal transduction) from Gene Operation (INO1001-0010MG, Ann Arbor, MI, USA) was used as a positive control drug. The antibodies included in this study were mouse monoclonal anti-α-smooth muscle actin (α-SMA, Clone 1A4; Sigma-Aldrich, St. Louis, MO, USA), rabbit polyclonal anti-cytokeratin (CK) 7 (17513-1-AP) and CK19 (10712-1-AP) (Proteintech Group Inc., Chicago, IL, USA), mouse monoclonal anti-hepatocyte nuclear factor 4 alpha (HNF-4α, clone H1; sc-374229; Santa Cruz Biotechnology, Inc. CA, USA), mouse monoclonal anti-Numb (Proteintech Group Inc.), rabbit polyclonal anti-Numb (Cat: YT5320, ImmunoWay Biotechnology Company, Newark, DE, USA), rabbit polyclonal anti-RBP-Jκ (Cat: 5313; Cell Signaling Technology, Danvers, MA, USA), Hes1 (ab108937; Abcam, Cambridge, UK), and mouse monoclonal anti-glyceraldehyde-3-phosphate dehydrogenase (GAPDH, Chemicon International, Billerica, MA, USA). IRDye 800CW donkey anti-mouse IgG (H + L) (LI-COR Bioscience, San Jose, CA, USA) and IRDye 680RD donkey anti-rabbit IgG (H + L) (LI-COR Bioscience, San Jose, CA, USA) were also purchased.

### BMSC isolation, culture, and identification

BMSC isolation was referenced Wang et al.^15^. In brief, Male Sprague–Dawley (SD) rats (80–100 g) were sacrificed under aseptic conditions via cervical dislocation, and the femur and tibia were removed. The ends of the femur or tibia were cut open to expose the medullary cavity and repeatedly washed with Dulbecco's Modified Eagle Medium (DMEM, Life Technologies, Gibco, Carlsbad, CA, USA). The cells were then collected and cultured in DMEM supplemented with 15% fetal bovine serum (FBS, Gibco) and 1% penicillin–streptomycin (Gibco) for 24 h. At 24 h, a half volume of medium was changed for fresh medium, with the medium completely replaced at 48 h. The BMSCs were then passaged every 3–5 d.

BMSC culture purity was determined by detecting CD10, CD14, CD29, CD34, CD45 and CD90 (BD Biosciences, San Jose, CA, USA) markers using FACScan (BD Biosciences)^16–18^, with isotypic antibodies serving as a control. In addition, the BMSC cell cycle was also detected.

### Adipogenic and Osteogenic induction

To assess the differentiation potential of the BMSCs, Adipogenic and Osteogenic induction referenced to reported method^15^. In briefly, cells were plated on Petri dishes in 15% FBS/DMEM-L. For induction of adipogenesis, when the cells fused 100%, add the lipogenic induction Asolution (Cyagen Biosciences Inc.containing1% penicillin–streptomycin, 1% glutamine, 0.2% insulin, 0.1% 3-isobutyl-1-methyl xanthine, 0.1% rosiglitazone, and 0.1% dexamethasone), replace it with the lipogenic induction B solution (Cyagen Biosciences Inc. containing 1% penicillin–streptomycin, 1% glutamine, and 0.2% insulin) three days later, and replace it with the lipogenic induction A solution one day later. Repeat this process. After 3 weeks of differentiation, cells were fixed and sliced. Adipogenic differentiation was monitored as red droplets after Oil Red O staining. For induction of osteogenic differentiation, when the cells fused 60%, the osteoblast induction solution (Cyagen Biosciences Inc. containing 1% penicillin–streptomycin, 1% glutamine, 0.2% ascorbiate, 0.2% β-glycerophosphate, and 0.01% dexamethasone) was added, and the fluid was changed every 3 days. After 4 weeks of differentiation, cells were fixed and sliced. The osteogenic-induced culture was analyzed by Alizarin Red staining for visualization of calcium deposition.

### BMSC *Numb* gene Knockdown

RNA interference (RNAi) was used to knockdown of BMSC *Numb* gene (BMSC^*Numb-*KD^). Lentiviral (LV) vectors were labeled with enhanced green fluorescent protein (EGFP). LV-*Numb*-RNAi (titer: 6 × 10^8^ TU/ml, Shanghai Genechem Co., Ltd., Shanghai, China) was transfected into P3 BMSCs using a multiplicity of infection (MOI) = 80 and with the addition of both polybrene and enhanced infection solution (ENi.S, Shanghai GeneChem Co., Ltd, Shanghai, China). The component sequence of LV-Numb-RNAi (52618-1) is hU6-MCS-Ubiquitin-EGFP-IRES-puromycin, and its target sequence is 5′-AAGAGAGGAGATCATGAAACA-3′. The negative control (NC) samples were transfected with CON077, a negative control lentivirus (titer: 8 × 10^8^ TU/ml, Shanghai Genechem Co., Ltd.), its component sequence is hU6-MCS-Ubiquitin-EGFP-IRES-puromycin, and the insertion sequence is 5′-TTCTCCGAACGTGTCACGT-3′. After transfection for 8–10 h, the media was replaced with vector-free medium.

### Animals and experimental protocol

Male SD rats (160–180 g) were purchased from Vital River Laboratory Animal Technology Co., Ltd. (Beijing, China). Animals were maintained in an environment with a constant temperature and supplied with laboratory chow and water ad libitum. The experimental protocols were approved by the Animal Research Committee at Shanghai University of TCM (No. PZSHUTCM18111607).

Bile duct ligation (BDL) with modifications was performed as previously described^19^. In brief, 42 rats were randomly divided into sham group (*n* = 6) and model group (*n* = 36). Model rats were anesthetized with pentobarbital sodium and laparotomy was performed with a sterile technique. The common bile duct and the left and right hepatic ducts were isolated. The left and right hepatic ducts and the hepatic portal and duodenal site of the common bile duct were ligated, respectively, and the abdomen was closed. In sham rats, the surgery was identical, except that the bile duct was not ligated. After the BDL, model rats were randomly divided into BDL (*n* = 6), HQD (*n* = 6), DAPT (*n* = 6), BMSC *Numb* empty virus (negative control, BMSC^NC-KD^, *n* = 6), BMSC *Numb* knockdown (BMSC^*Numb*-KD^, *n* = 6), and HQD plus BMSC^*Numb*-KD^ (*n* = 6) groups, and single injection of 1 × 10^6^ BMSC^NC-KD^ cells or BMSC^*Numb*-KD^ cells into livers of corresponding groups. From the second week after BDL, HQD, HQD plus BMSC^*Numb*-KD^ and DAPT groups were administrated orally at dosages of 0.935 g/kg HQD or 50 mg/kg DAPT respectively for 3 weeks once per day. Sham and BDL rats were given same volume of physiological saline. At the end of 4 weeks, all rats were euthanized with pentobarbital sodium at a dose of 60 mg/kg, and blood and hepatic tissue samples were obtained.

### Histopathological and immunohistochemical analyses

Liver histopathological changes were determined with paraffin-embedded liver sections which is stained with hematoxylin and eosin (H&E) or 0.1% (w/v) Sirius Red (Direct Red 80; Aldrich, Milwaukee, WI, USA). Immunohistochemistry was performed using paraffin-embedded sections as described previously^20^. In brief, sections were deparaffinized, washed, and pre-incubated in blocking solution prior to incubating with primary antibodies, including mouse monoclonal anti-α-SMA (1:200), Cytokeratin7 (CK7, 1:100), CK19 (1:100) and Hepatocyte Nuclear Factor 4 alpha (HNF4α, 1:100). Sections were then incubated with HRP-conjugated secondary antibodies (1:1,000) and washed. The samples were visualized using DAB, with hematoxylin counterstaining, and imaged with a Leica SCN400 scanner (Leica Microsystems Inc., Concord, ON, Canada).

For Immunofluorescent staining, frozen specimens were cut into 7-μm-thick sections and immunofluorescently stained to detect co-expression of EGFP (*marked lentivirus*) and CK7 (1:50) or EGFP and CK19 (1:50). Additionally, Numb (1:50), RBP-Jκ (1:1,000) and Hes1 (1:100) were also examined. After incubation with the primary antibodies, samples were washed with PBST, and incubated with Alexa Fluor 488 goat anti-mouse IgG (A11001; Invitrogen, Carlsbad, CA, USA) or Alexa Fluor 594 goat anti‐rabbit IgG (AB6939; Abcam, Cambridge, UK) secondary antibodies. The nucleus were stained with 4′,6-diamidino-2-phenylindole (DAPI; 1:1,000) and images were obtained by a confocal laser scanning microscope FV10i (Olympus, Japan).

### Hepatic hydroxyproline content

Hepatic hydroxyproline (Hyp) was determined with a modified method of Jamall et al.^21^. In brief, liver tissues were homogenized and hydrolyzed in 6 N HCl at 110 °C for 18 h. After filtration of the hydrolysate through a 0.45-mm Millipore filter (Millipore, Bedford, MA, USA), chloramine T was added (final concentration 2.5 mM). The mixture was then treated with 410 mM paradigm ethylamino-benzaldehyde and incubated at 60 °C for 30 min. After cooling to room temperature, samples were read at 560 nm against a reagent blank, which contained the complete system without added tissue. Hyp was quantified from a standard substance (Nakateyitesuku Company, Japan).

### Immunoblot analysis

Liver tissue was lysed in RIPA buffer containing a mixture of protease inhibitor and phosphatase inhibitor and then homogenized in ice-cold water. Protein concentrations were determined using a bicinchoninic acid (BCA) protein assay kit (Thermo). Total protein (25 μg) was resolved by SDS-PAGE, transferred onto PVDF membranes, blocked with 5% (w/v) Bovine serum albumin (BSA) solution, and incubated with the following primary antibody: α-SMA (1:1,000), CK7 (1:1,500), CK19 (1:1,500), Numb (1:300), RBP-Jκ (1:1,000), Hes1(1:500), and GAPDH (1:10,000). The following second antibodies were also used: IRDye 800CW Donkey anti-Mouse IgG (H + L) (1:10,000) and IRDye 680RD Donkey anti-Rabbit IgG (H + L) (1:1,000). Finally, the data were analyzed by Odyssey 2.1 software.

### Quantitative real-time PCR analysis

mRNA expressions of α-SMA, Collagen type I (ColI), ColIV, tumor necrosis factor-alpha (TNF-α), transforming growth factor-beta (TGF-β) 1, Notch-1/-2-/3/-4, Jagged (JAG) -1/-2, Delta like (DLL)-1/-4, Numb, Hes1, RBP-Jκ, ligand of Numb proteins X (LNX)-1/-2, itchy E3 ubiquitin protein ligase (ITCH), CK7, and CK19 were assessed by quantitative real-time PCR (qRT-PCR). Total RNA was isolated from frozen hepatic tissues using Isogen (TOYOBO, Kita-ku, Osaka, Japan)^22^. Each sample was reverse-transcribed using Super Script II Reverse Transcriptase (Thermo Fisher Scientific, Waltham, MA, USA). The samples were then analyzed using fluorescence-based qRT-PCR and SYBR Green Real-Time PCR Master Mix (TOYOBO) according to the manufacture’s protocols. Primers and oligonucleotide probes were designed using Primer Express (Sigma Chemical), and are listed in Table 1. Each PCR amplification was performed on five rats in both experimental and control groups. Individual gene expression was normalized by GAPDH. The conditions for the One-Step SYBR RT-PCR (Perfect Real Time) were as follows: an initial step of 15 min at 42 °C, 2 min at 95 °C, and then 40 amplification cycles of denaturation at 95 °C for 15 s, and annealing and extension at 60 °C for 1 min.

**Table 1:** Primer pairs and probes used for real-time PCR.

Gene		Primer sequence	Note
*Notch1*	Forward Reverse	TGGATGAGGAAGACAAGCATTA GAAAAGCCACCGAGATAGTCAG	*SYBR*
*Notch2*	Forward Reverse	GAG GAA GAA GTG TCT CAA GTG GCA TCA GAA ACA TAT G	*SYBR*
*Notch3*	Forward Reverse	GAC AAG GAC CAC TCC CAC TACT ATC CAC ATC ATC CTC ACA ACT G	*SYBR*
*Notch4*	Forward Reverse	TGT CAG GAA CCA GTG TCA GAA C CCT GGG CTT CAC ATT CAT CTA T	*SYBR*
*Jagged1*	Forward Reverse	CCA TCA AGG ATT ATG AGA AC TGG TGC TTA TCC ATA TCA	*SYBR*
*Jagged2*	Forward Reverse	AAA TGA GTG GTC CGT GGC AGA TGG TTG GAA GCC TTG TCT GCT	*SYBR*
*Delta 1*	Forward Reverse	GTG TGC AGA TGG TCC TTG CTT C CTG ACA TCG GCA CAG GTA GGA G	*SYBR*
*Delta 4*	Forward Reverse	GCA GAA CCA CAC ACT GGA CTA T TGG CAC CTT CTC TCC TAA ACT C	*SYBR*
*Numb*	Forward Reverse	GCT ACT TTC GAT GCC AGT AGA ACC A CTG TTG CCA GGA GCC ACT GA	*SYBR*
*Hes1*	Forward Reverse	GAC GGC CAA TTT GCT TTC GAC ACT GCG TTA GGA CCC	*SYBR*
*RBP-Jκ*	Forward Reverse	TTG CTT ACC TTC AGG CGT GTG GCC CAA TGA GTC TGC TGC AA	*SYBR*
*α-SMA*	Forward Reverse	AAT GGC TCT GGG CTC TGT AA TCT CTT GCT CTG GGC TTC AT	*SYBR*
*Collagen I*	Forward Reverse	ACG TCC TGG TGA AGT TGG TC TCC AGC AAT ACC CTG AGG TC	*SYBR*
*CollagenIV*	Forward Reverse	TTT CCA GGG TTA CAA GGT GT AGT CCA GGT TCT CCA GCA TC	*SYBR*
*TGF-β1*	Forward Reverse	ATT CCT GGC GTT ACC TTG G AGC CCT GTA TTC CGT CTC CT	*SYBR*
*TNF-α*	Forward Reverse	GAC GTG GAA CTG GCA GAA GAG TTG GTG GTT TGT GAG TGT GAG	*SYBR*
*CK7*	Forward Reverse	AGG AAC AGA AGT CAG CCA AGA G GCA ACA CAA ACT CAT TCT CAG C	*SYBR*
*CK19*	Forward Reverse	GAT CTG CGT AGT GTG G AAA ACC AAA CTG GGG ATG	*SYBR*
*LNX1*	Forward Reverse	TGC TGC CAG GAG ACA TCA T CAT TGC TTC TGC TAC GGA ACT T	*SYBR*
*LNX2*	Forward Reverse	ACA CAG ATT GAG GGT GAA ACT GGT CCA CAC AGG AAG AGG T	*SYBR*
*ITCH*	Forward Reverse	ATG GGA GAT TTG TCA GTT TGT C CAG CGT CAT TCT GTG TAG CA	*SYBR*
*GAPDH*	Forward Reverse	GGC ACA GTC AAG GCT GAG AAT G ATG GTG GTG AAG ACG CCA GTA	*SYBR*

### WB-F344 cell line culture and treatment

In vitro studies were performed using the WB-F344 cell line, a rat hepatic progenitor cell line^23^. The cells were cultured in Ham’s F12 medium (Life Technologies) supplemented with 10% fetal calf serum (Gibco). Chemically induced differentiation was obtained by culturing WB-F344 cells on six-well Permanox Lab-Tek culture slides (Nalge Nunc International, Naperville) at a density of 2 × 10^3^ cells/cm^2^, starting 24 h after seeding. Cells were divided into normal group (N), SB group (3.75 mM, Sigma, B5887-1G)^24^, SB plus HQD (800 μg/ml) group , SB plus DAPT (50 μM) group, SB plus LV empty virus (negative control, NC) group, SB plus *Numb* KD group, and SB plus HQD plus *Numb* KD group (*n* = 3, respectively). RNAi was performed as described above, but at a MOI = 50. At 6 h post-transfection, the media was replaced with media containing SB, with or without HQD, while positive control sample media contained SB and DAPT without HQD. Media was changed every 2 days, and the culture time was 7 days.

### Statistical analysis

All data are presented as a mean ± SD. Statistical analyses were performed using an analysis of variance (ANOVA) for multiple comparisons with SPSS 10.0 software and *P* < 0.05 was considered statistically significant.

### Ethics approval and consent to participate

The experimental protocol was approved by the Animal Research Committee at Shanghai University of Traditional Chinese Medicine (No. PZSHUTCM18111607). All methods were performed in accordance with relevant guideline and regulations in the manuscript.

## Results

### Lentivirus-mediated knockdown of the BMSC *Numb* gene

P3 BMSC morphological characteristics were consistent with long fusiform and whirl-like growth. BMSCs were confirmed by flow cytometry, and showed that CD10 (−), CD14 (−), CD29 (+), CD34 (−), CD45 (−), and CD90 (+) (Fig. 1a). Furthermore, the proliferative capacity was evaluated by examining the cell cycle via flow cytometry, and showed that 72.36% of the BMSCs were in the G1 phase (Fig. 1b). Additionally, the BMSCs showed osteogenic and adipogenic abilities following the differentiation assay, with a large number of calcium deposits following osteogenic induction, and a large number of fat droplets following adipogenic induction (Fig. 1c,d). These results demonstrate that the obtained BMSCs have strong differentiation ability. Next, cells were transfected with lentivirus and reached a transfection rate of more than 80% at a MOI = 80, and maintained a normal cell morphology (Fig. 1e). The qRT-PCR result showed that *Numb* gene expression was significantly decreased in the *Numb*-KD group compared to that in the negative control (NC) group (*P* < 0.01), about 33% that of the NC group (Fig. 1f). In addition, the Numb protein expression was significantly decreased in the *Numb*-KD group compared to that in the NC group (*P* < 0.01) (Fig. 1g,h).

### BMSC^***Numb***-KD^ transplantation reduces the effect of HQD against CLF

**Figure 2:**
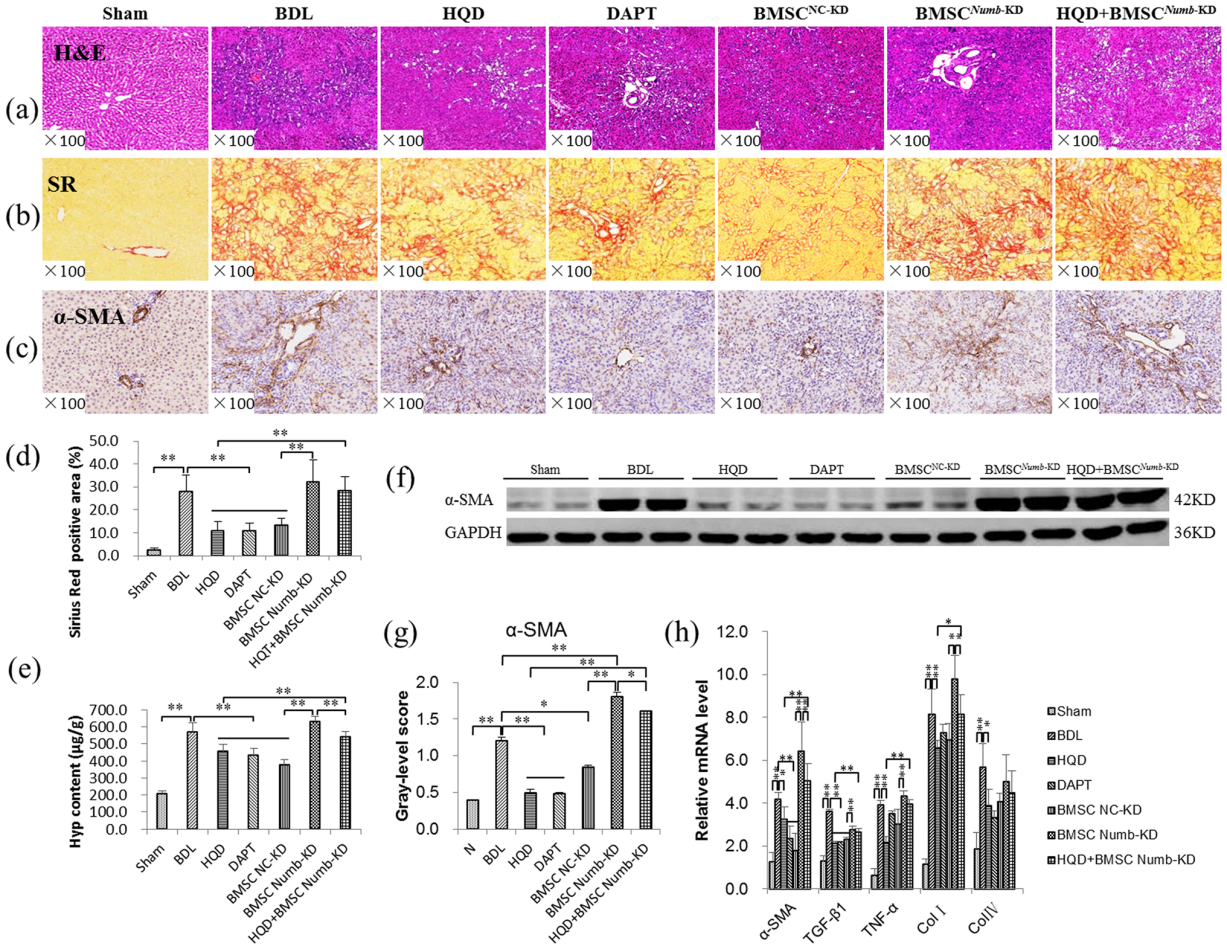
BMSC^*Numb*-KD^ transplantation reduces the anti-fibrotic effect of HQD. (**a**) H&E staining (×100). (**b**) Sirius Red staining (×100). (**c**) α-SMA immunostaining (×200). (**d**) Positive area percentage of Sirius Red staining (*n* = 5 per group). (**e**) Hyp content in liver tissue (*n* = 5 per group). (**f**) Immunoblotting for α-SMA, and (**g**) The gray-level score indicates the histogram of immunoblotting for α-SMA (*n* = 5 per group). (**h**) Relative mRNA levels of α-SMA, ColI, ColIV, TGF-β1 and TNF-α measured by qRT-PCR, mRNA levels were normalized by GAPDH (*n* = 5 per group). *P < 0.05, **P < 0.01. Sham, sham group; BDL, bile duct ligation group; HQD, Huang Qi decoction group; DAPT, DAPT group; BMSC^NC^, BMSC negative control group; BMSC^*Numb*-KD^, BMSC *Numb* knockdown group; and HQD + BMSC^*Numb*-KD^, HQD plus BMSC^*Numb*-KD^ group.

H&E staining showed extensive bile duct proliferation in the BDL and BMSC^*Numb*-KD^ groups, while bile duct proliferation was markedly reduced in the HQD and HQD plus BMSC^*Numb*-KD^ groups compared to that in the BDL and BMSC^*Numb*-KD^ groups, respectively. However, bile duct proliferation was markedly increased in the HQD plus BMSC^*Numb*-KD^ group compared to that in the HQD group (Fig. 2a).

Sirius red staining revealed that proliferated BECs were surrounded by abundant collagen in the BDL and BMSC^*Numb*-KD^ groups, while collagen deposition was markedly reduced in the HQD and HQD plus BMSC^*Numb*-KD^ groups compared to that in the BDL and BMSC^*Numb*-KD^ groups, respectively. However, collagen deposition levels were higher in the HQD plus BMSC^*Numb*-KD^ group compared to that in the HQD group (Fig. 2b). Moreover, immunostaining showed that α-SMA (myofibroblast marker) expression was localized in the fibrotic septa, which was consistent with that of the Sirius Red staining (Fig. 2c).

Sirius Red stained area was decreased significantly in HQD, DAPT and BMSC ^NC-KD^ groups compared to that in BDL group (*P* < 0.01). Sirius Red stained area in HQD plus BMSC ^*Numb*-KD^ group was decreased compared to that in BMSC^*Numb*-KD^ group but no significant difference (*P* > 0.05). However, Sirius Red stained area was significantly higher than that in HQD group (*P* < 0.01) (Fig. 2d).

As show in Fig. 2e, the Hyp content of liver tissues were significantly increased in the BDL and BMSC^*Numb*-KD^ groups compared to that in the Sham group (*P* < 0.01) and the BMSC^NC^ group (*P* < 0.01), respectively, while it was significantly decreased in the HQD and HQD plus BMSC^*Numb*-KD^ groups compared to that in the BDL group (*P* < 0.01) and the BMSC^*Numb*-KD^ group (*P* < 0.01), respectively. However, Hyp content was higher in the HQD plus BMSC^*Numb*-KD^ group compared to that in the HQD group (*P* < 0.01). Moreover, the mRNA and protein levels of α-SMA were consistent with the changes of Sirius Red standing positive area and Hyp content (Fig. 2f,g).

Additionally, the expression levels of TGF-β1, TNF-α, ColI and ColIV mRNA were significantly increased in the BDL and BMSC^*Numb*-KD^ groups compared to that in the Sham group (*P* < 0.01) and BMSC^NC^ group (*P* < 0.05 or *P* < 0.01), respectively, while the expression levels were significantly reduced in the HQD group compared to that in the BDL group (*P* < 0.05 or* P* < 0.01), and α-SMA and ColI expression was significantly reduced in the HQD plus BMSC^*Numb*-KD^ group compared to that in the BMSC^*Numb*-KD^ group (*P* < 0.05). However, the α-SMA, TGF-β1, TNF-α, and ColI levels were significantly increased in the HQD plus BMSC^*Numb*-KD^ group compared to that in the HQD group, (*P* < 0.05 or *P* < 0.01) (Fig. 2h).

### BMSC^***Numb***-KD^ transplantation reduces HQD inhibition on bile duct proliferation

**Figure 3:**
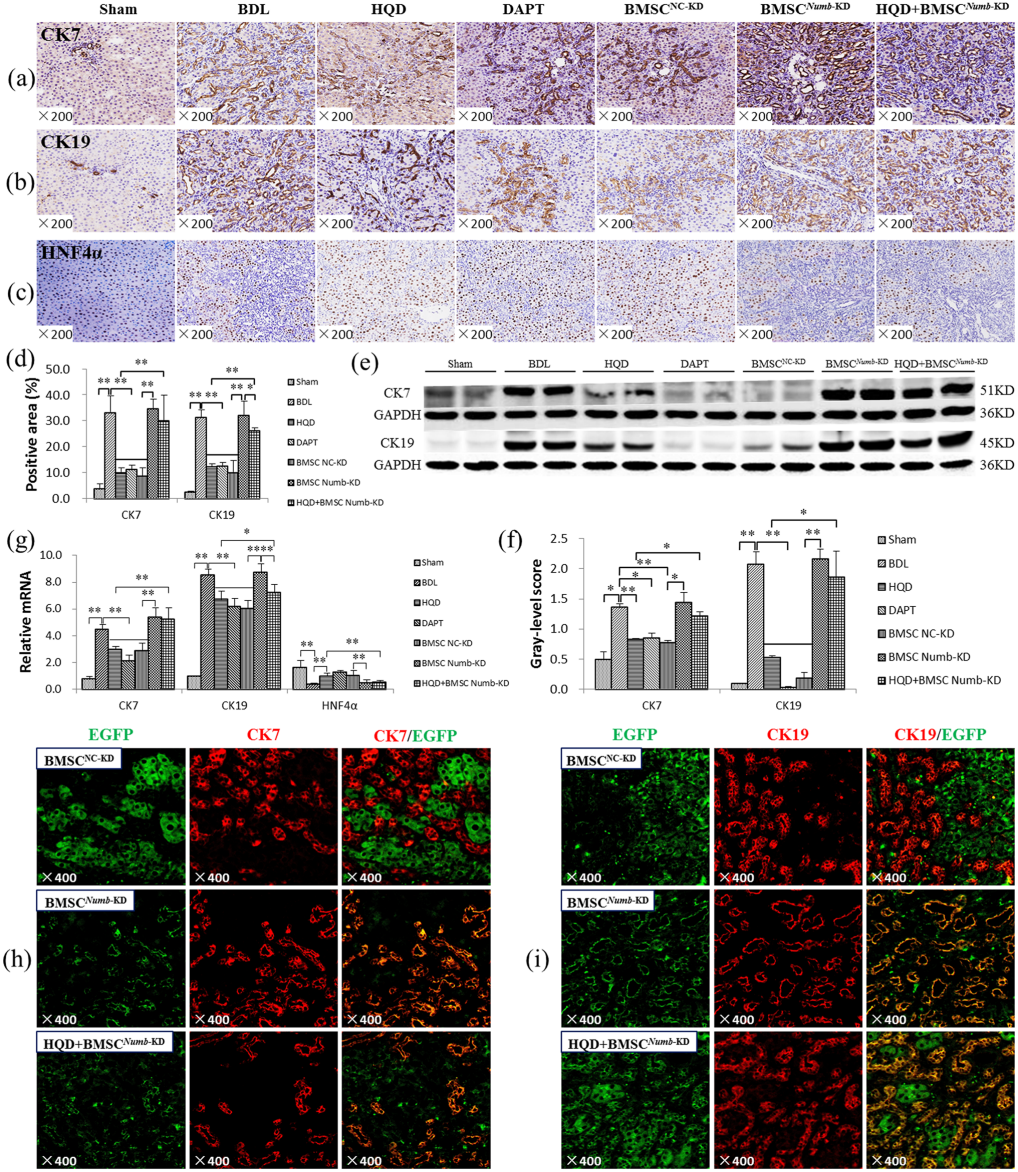
BMSC^*Numb*-KD^ transplantation reduces the inhibitory effect of HQD on bile duct proliferation. (**a**) Immunostaining of CK7 (×200). (**b**) Immunostaining of CK19 (×200). (**c**) Immunostaining of HNF4α (×200). (**d**) Positive area percentage of CK7 and CK19 immunostaining (*n* = 5 per group). (**e**) Immunoblotting of CK7 and CK19, and (**f**) The gray-level score indicates the histogram of immunoblotting for CK7 and CK19 (*n* = 5 per group). (**g**) Relative CK19, CK7 and HNF4α expression levels measured by qRT-PCR, mRNA levels were normalized by GAPDH (*n* = 5 per group). (**h**) Double immune-staining of CK7 (red) and EGFP (green) (×400). (**i**) Double immune-staining of CK19 (red) and EGFP (green) (×400). *P < 0.05, **P < 0.01. Sham, sham group; BDL, bile duct ligation group; HQD, Huang Qi decoction group; DAPT, DAPT group; BMSC^NC^, BMSC negative control group; BMSC^*Numb*-KD^, BMSC *Numb* knockdown group; and HQD + BMSC^*Numb*-KD^, HQD plus BMSC^*Numb*-KD^ group.

CK7 and CK19 are regarded as a hallmark of BEC^25^, immunostaining showed that CK7 and CK19 were mainly expressed in BECs in the Sham and BMSC^NC^ group. In BDL and BMSC^*Numb*-KD^ rats, CK7 and CK19 were strongly expressed in proliferated BECs, while they were clearly decreased in the HQD and HQD plus BMSC^*Numb*-KD^ groups compared with the BDL and BMSC^*Numb*-KD^ groups, respectively. However, in the HQD plus BMSC^*Numb*-KD^ group, CK7 and CK19 were increased relative to the HQD group (Fig. 3a,b).

It is consistent with immunostaining, as shown in Fig. 3d, the positive areas of CK7 and CK19 were increased significantly in the BDL and BMSC^*Numb*-KD^ groups compared to that in Sham (*P* < 0.01) and BMSC^NC^ (*P* < 0.01) groups, respectively, while the positive area of CK19 was decreased significantly in the HQD and the HQD plus BMSC^*Numb*-KD^ groups compared to that in the BDL (*P* < 0.01) and BMSC^*Numb*-KD^ (*P* < 0.05) groups, respectively; the expression of CK7 were decreased significantly in the HQD group compared with the BDL (*P* < 0.01), and CK7 showed a decreasing trend in the HQD plus BMSC^*Numb*-KD^ group compared to that in the BMSC^*Numb*-KD^ group, with no significant difference (*P* > 0.05). However, in the HQD plus BMSC^*Numb*-KD^ group, the positive areas of CK7 and CK19 were significantly increased compared to that in the HQD group (*P* < 0.01).

In addition, the protein expression levels of CK7 and CK19 were increased significantly in the BDL and BMSC^*Numb*-KD^ groups compared to that in the Sham (*P* < 0.05 or *P* < 0.01) and BMSC^NC^ (*P* < 0.05 or *P* < 0.01) groups, respectively, while the protein expression levels were decreased significantly in the HQD group compared to that in the BDL group (*P* < 0.01). However, they were increased significantly in the HQD plus BMSC^*Numb*-KD^ group compared to that the HQD group (*P* < 0.05) (Fig. 3e,f). Additionally, CK7 and CK19 mRNA expression was consistent with their protein expression (Fig. 3g).

HNF4α, a key regulator of hepatocyte differentiation, which also maintains a mature hepatocyte differentiation phenotype during embryonic development^26,27^, was also examined. Immunostaining showed that in the BDL and BMSC^*Numb*-KD^ groups, HNF4α expression was clearly lower compared to that in the Sham and BMSC^NC^ groups, respectively, while its expression was increased in the HQD and HQD plus BMSC^*Numb*-KD^ groups compared to that in the BDL and BMSC^*Numb*-KD^ groups, respectively. However, HNF4α expression was decreased in the HQD plus BMSC^*Numb*-KD^ group compared to that in the HQD group in immunostaining (Fig. 3c). Moreover, the HNF4α mRNA level was consistent with the immunostaining changes (Fig. 3g).

To further evaluate BMSC^*Numb*-KD^ differentiation in liver, co-immunostaining with EGFP/CK19 or EGFP/CK7 was performed. The result showed that no clearly co-expression was noted in the BMSC^NC^ group, while extensive co-expression was noted in proliferating BECs in the BMSC^*Numb*-KD^ group, and the above pathological change was not clearly reversed in the HQD plus BMSC^*Numb*-KD^ group (Fig. 3h,i). These results suggest that BMSCs differentiate into BECs after *Numb* knockdown in liver, and that HQD has no obvious intervention effect on this pathological process.

### BMSC^***Numb***-KD^ transplantation reduces the effect of HQD inhibition of Notch signaling activation

**Figure 4:**
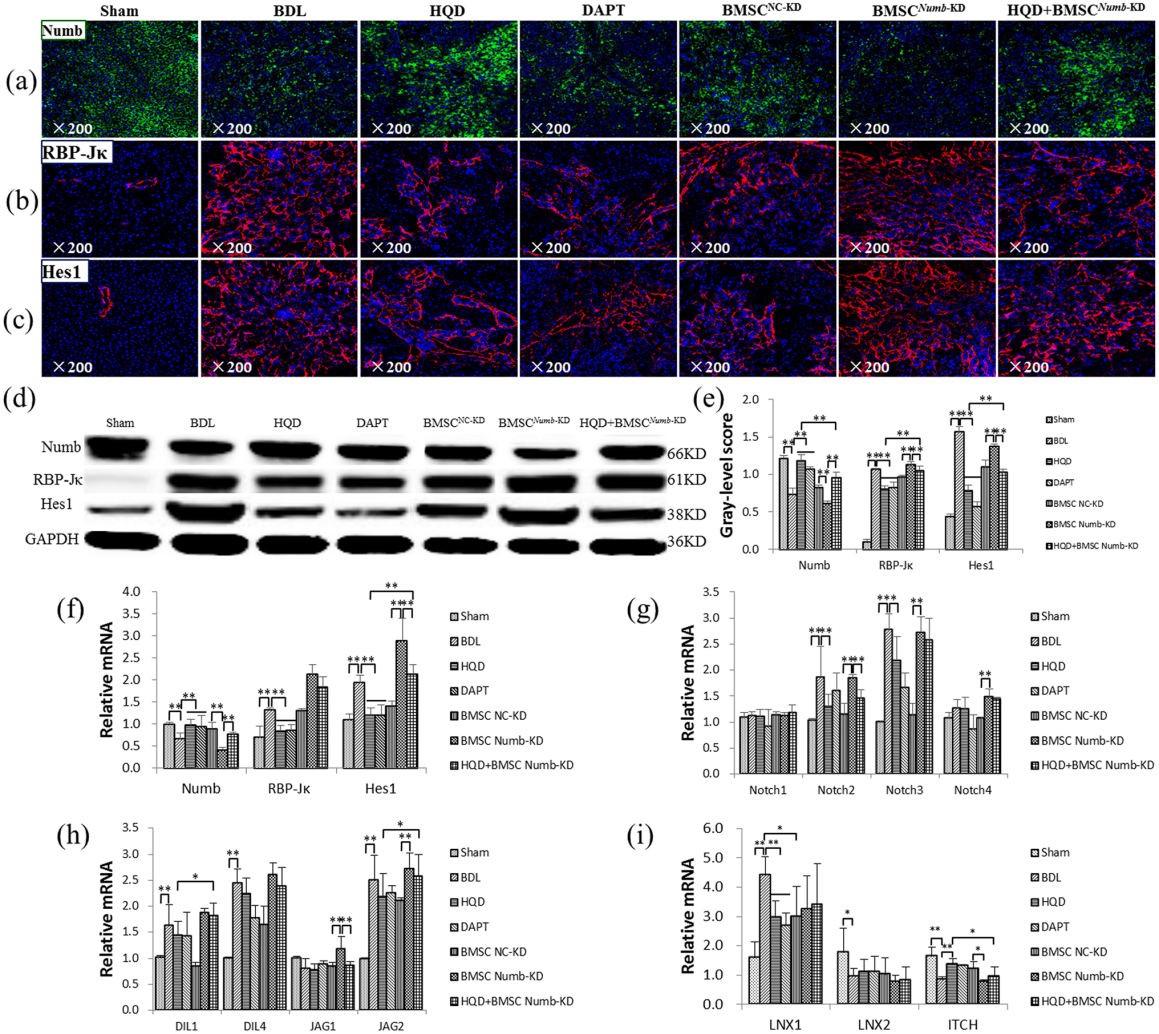
BMSC^*Numb*-KD^ transplantation reduces the inhibitory effect of HQD on Notch signaling activation. (**a**) Immunostaining of Numb (×200). (**b**) Immunostaining of RBP-Jκ (×200). (**c**) Immunostaining of Hes1 (×200). (**d**) Immunoblotting for Numb, RBP-Jκ and Hes1, and (**e**) The gray-level score indicates the histogram of immunoblotting for Numb, RBP-Jκ and Hes1 (*n* = 5 per group). (**f**) Relative mRNA levels of Numb, RBP-Jκ and Hes1. (**g**) Relative mRNA levels of Notch-1, -2, -3 and -4. (**h**) Relative mRNA levels of DLL1, DLL4, Jag1 and Jag2. (**i**) Relative mRNA levels of LNX1, LNX2 and ITCH. All mRNA levels were normalized by GAPDH (*n* = 5 per group). *P < 0.05, **P < 0.01.Sham, sham group; BDL, bile duct ligation group; HQD, Huang Qi decoction group; DAPT, DAPT group; BMSC^NC^, BMSC negative control group; BMSC^*Numb*-KD^, BMSC *Numb* knockdown group; and HQD + BMSC^*Numb*-KD^, HQD plus BMSC^*Numb*-KD^ group.

As shown in Fig. 4a–c, immunofluorescence staining clearly indicated that Numb was localized in hepatocytes, while RBP-Jκ and Hes1 were associated with proliferating BECs. In the BDL and BMSC^*Numb*-KD^ groups, Numb expression was markedly decreased, while RBP-Jκ and Hes1 levels were markedly increased compared to that in the Sham and BMSC^NC^ groups, respectively. In the HQD and HQD plus BMSC^*Numb*-KD^ groups, Numb expression was increased, while RBP-Jκ and Hes1 expressions were decreased compared to that in BDL and BMSC^*Numb*-KD^ groups, respectively. However, the improvement in HQD group was better than that of HQD plus BMSC^*Numb*-KD^ group.

Additionally, Numb, RBP-Jκ and Hes1 protein and mRNA levels were also examined. In the BDL and BMSC^*Numb*-KD^ groups, Numb was significantly decreased (*P* < 0.01), while RBP-Jκ and Hes1 were markedly increased compared to that in the Sham and BMSC^NC^ groups (*P* < 0.01), respectively. In the HQD and HQD plus BMSC^*Numb*-KD^ groups, Numb protein and mRNA expression levels were significantly increased, while RBP-Jκ and Hes1 expression levels were markedly decreased compared to that in the BDL and BMSC^*Numb*-KD^ groups (*P* < 0.05 or *P* < 0.01), respectively. However, in the HQD group, Numb protein and mRNA expression levels were significantly increased, while RBP-Jκ and Hes1 expression levels were markedly decreased compared to that in the HQD plus BMSC^*Numb*-KD^ (*P* < 0.05 or *P* < 0.01) (Fig. 4d–f).

To further characterize the effect of *Numb* knockdown in BMSCs, the upstream components of Notch signaling were also examined. The results showed that Notch-2/-3, DLL1, and Jag-1/-2 mRNA expression levels were significantly increased in the BDL group compared to that in the Sham group (*P* < 0.01), while Notch-2/-3 and Jag-1/-2 were significantly decreased in the HQD group compared to that in the BDL group (*P* < 0.05 or *P* < 0.01). In the BMSC^*Numb*-KD^ group, Notch-2/-3/-4, DLL-1/-4 and Jag-1/-2 were significantly increased relative to the BMSC^NC^ group (*P* < 0.01). However, only Notch-2 and DLL-4 were significantly decreased in the HQD plus BMSC^*Numb*-KD^ group compared to that in the BMSC^*Numb*-KD^ group (*P* < 0.01) (Fig. 4g,h). Suggesting that *Numb* knockdown promotes the activation of Notch pathway in BMSCs, while decreases the inhibitory effect of HQD on Notch signal pathway.

Moreover, the mRNA expression of E3 ubiquitin ligase including LNX-1/-2 (promoting proteasome-dependent degradation of Numb^28^), and ITCH (promoting ubiquitination-dependent proteasomal degradation of the NICD^29^) were examined, the results showed that in the BDL and BMSC^*Numb*-KD^ groups, LNX1 was increased significantly, while the LNX2 and ITCH were decreased significantly compared to that in the Sham and BMSC^NC^ groups (*P* < 0.05 or *P* < 0.01), respectively. In contrast, the mRNA level of LNX1 was decreased significantly, while ITCH was significantly increased in the HQD group compared to that in the BDL group (*P* < 0.01). However, they were no change in HQD plus BMSC^*Numb*-KD^ group compared to that in the BMSC^*Numb*-KD^ group (*P* > 0.05). In addition, the mRNA expression of ITCH was significantly decreased in HQD plus BMSC^*Numb*-KD^ group compared to that in the HQD group (*P* < 0.05) (Fig. 4i).

### *Numb* knockdown reduces the inhibitory effect of HQD on WB-F344 differentiation into cholangiocyte phenotype in vitro

**Figure 5:**
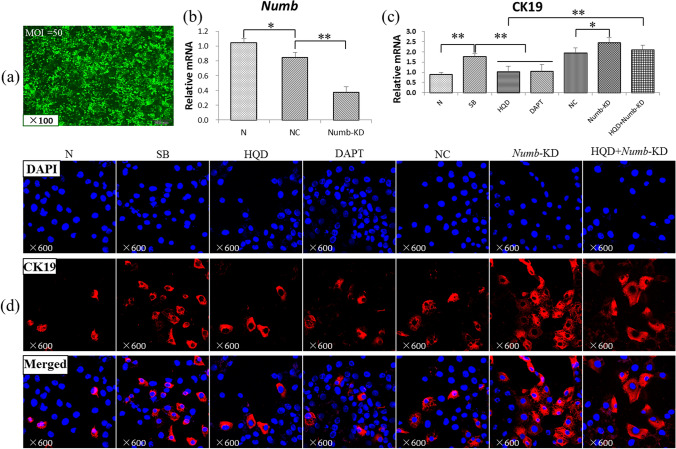
*Numb* knockdown reduces the inhibitory effect of HQD on WB-F344 differentiation into cholangiocyte phenotype. (**a**) Lentivirus transfected WB-F344. (**b**) Relative *Numb* level measured by qRT-PCR. (**c**) Relative CK19 level measured by qRT-PCR. All mRNA levels were normalized by GAPDH (*n* = 3 per group). (**d**) Immunostaining of CK19 (×600). *P < 0.05, **P < 0.01. N, normal group; SB, sodium butyrate group; HQD, SB plus Huang Qi Decoction group; DAPT, SB with DAPT group; NC, empty virus with SB group; *Numb*-KD, *Numb* knockdown with SB group; and HQD + *Numb*-KD, *Numb* knockdown and SB with Huang Qi Decoction group.

To further investigate the role of *Numb* in HQD against CLF, WB-F344 cells were utilized for further validation. The lentivirus transfection results showed that the cell transfection rate could reach more than 80% at a MOI = 50, with a normal cell morphology (Fig. 5a). Furthermore, the qRT-PCR result showed that Numb expression was significantly decreased in the *Numb*-KD group compared to that in the negative control (NC) group (*P* < 0.01; Fig. 5b).

Immunostaining showed that CK19 expression was markedly increased in the SB and *Numb*-KD groups compared to that in the N and NC groups, respectively. However, in the HQD group, CK19 expression was reduced compared to that in the SB group, but it was no clearly difference between the HQD plus *Numb*-KD and *Numb*-KD groups (Fig. 5d). Additionally, CK19 mRNA expression was significantly increased in the SB group (*vs.* N group, *P* < 0.01), while it was significantly decreased after HQD treatment (*vs.* SB group, *P* < 0.01). However, in the *Numb*-KD group, CK19 expression was significantly increased compared to that in the NC group (*P* < 0.05), but it was no significant difference between the HQD plus *Numb*-KD and *Numb*-KD groups. Furthermore, CK19 mRNA level was significantly higher in the HQD plus *Numb*-KD group compared to that in the HQD group (*P* < 0.01) (Fig. 5c). These findings suggest that *Numb* knockdown significantly attenuates the inhibitory effect of HQD on WB-F344 cell differentiation into BECs phenotype.

### *Numb* gene knockdown reduces the inhibitory effect of HQD on Notch signaling activation of WB-F344 in vitro

**Figure 6:**
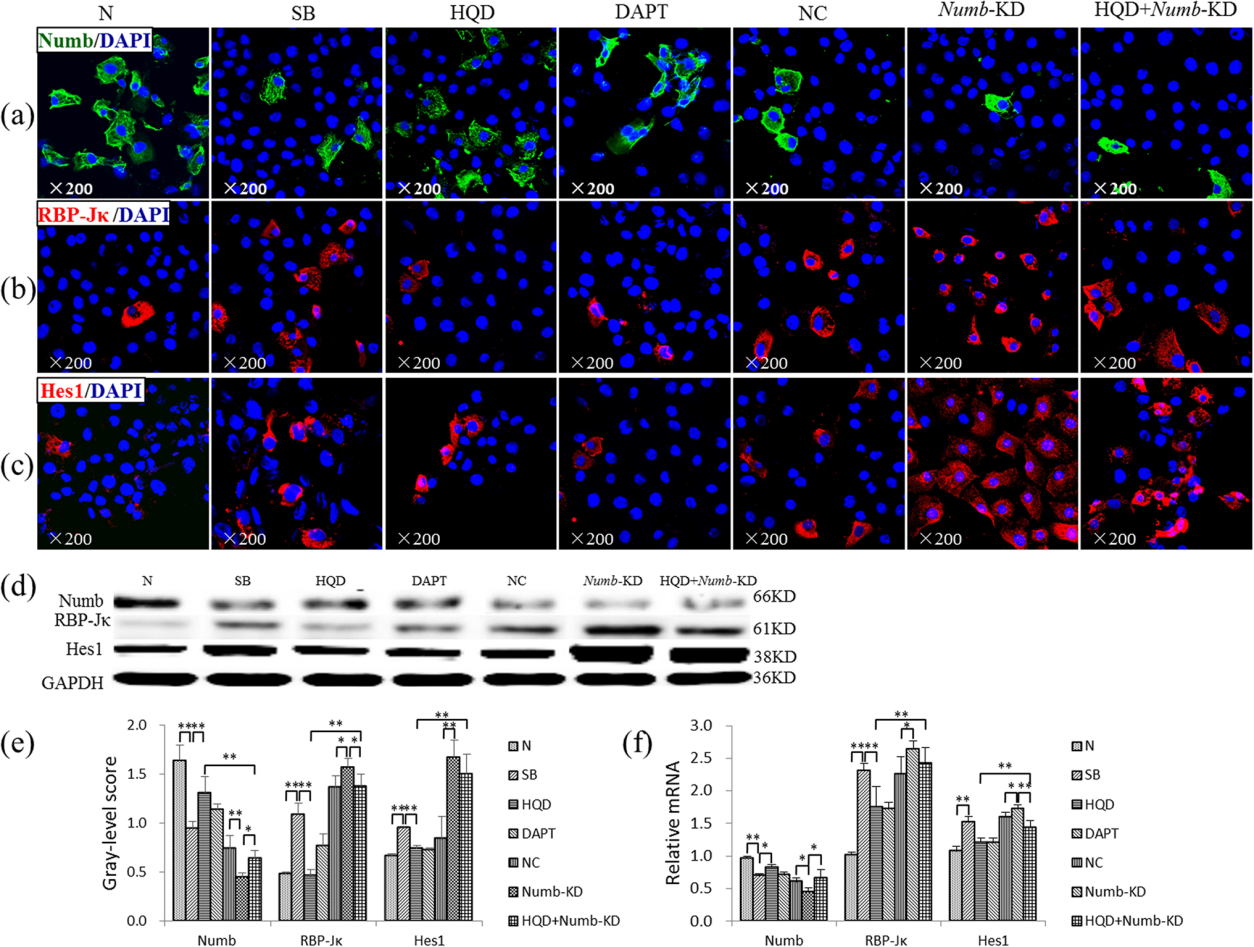
*Numb* knockdown reduces the inhibitory effect of HQD on Notch signal pathway activation in WB-F344. (**a**) Immunostaining of Numb (×600). (**b**) Immunostaining of RBP-Jκ (×600). (**c**) Immunostaining of Hes1 (×600). (**d**) Immunoblotting for Numb, RBP-Jκ and Hes1, and (**e**) The gray-level score indicates the histogram of immunoblotting for Numb, RBP-Jκ and Hes1 (*n* = 3 per group). (**f**) Relative levels of Numb, RBP-Jκ and Hes1 measured by qRT-PCR, and mRNA levels were normalized by GAPDH (*n* = 3 per group). *P < 0.05, **P < 0.01. N, normal group; SB, sodium butyrate group; HQD, SB plus Huang Qi decoction group; DAPT, SB with DAPT group; NC, empty virus with SB group; *Numb*-KD, *Numb* knockdown with SB group; and HQD + *Numb*-KD, *Numb* knock down and SB with Huang Qi Decoction group.

As shown in Fig. 6a–c, immunostaining showed that Numb expression was decreased in the SB and *Numb*-KD groups compared to that in N and NC groups, respectively, with the greatest reduction noted in the *Numb*-KD group. On the other hand, RBP-Jκ and Hes1 expression were markedly increased in the SB and *Numb*-KD groups compared to that in the N and NC groups, respectively. In the HQD and HQD plus *Numb*-KD groups, Numb expression was markedly increased, while RBP-Jκ and Hes1 were markedly decreased relative to the SB and *Numb*-KD groups, respectively. However, the improvement degree of compared to that in HQD group was better than that of HQD plus *Numb*-KD group.

Immunoblotting showed that the protein expression level of Numb was decreased significantly in the SB and *Numb*-KD groups compared to that in the N and NC groups (*P* < 0.01), respectively. Additionally, RBP-Jκ and Hes1 protein levels were significantly increased in the SB and *Numb*-KD groups compared to that in the N and NC groups (*P* < 0.05 or *P* < 0.01), respectively. When compared to that in the SB and *Numb*-KD groups, Numb protein levels were significantly increased in the HQD group (*P* < 0.01) and HQD plus *Numb*-KD group (*P* < 0.05), respectively, with the HQD group is of significantly higher levels compared to that in the HQD plus *Numb*-KD group (*P* < 0.01). Furthermore, RBP-Jκ and Hes1 levels were significantly decreased in the HQD and HQD plus *Numb*-KD groups compared to that in the SB and *Numb*-KD groups (*P* < 0.05 or *P* < 0.01), respectively, with significantly higher levels noted in the HQD plus *Numb*-KD group compared to that in the HQD group (*P* < 0.01) (Fig. 6d,e). Additionally, Numb, RBP-Jκ and Hes1 mRNA expression levels were consistent with their corresponding protein levels (Fig. 6f). These results suggest that *Numb* knockdown reduces the inhibitory effect of HQD on Notch signaling activation in WB-F344 cells.

## Discussion

### BMSC^***Numb***-KD^ transplantation promotes CLF progression

In recent years, Numb, a negative regulator of Notch signaling, has been widely examined in the science, with Numb found to modulate Notch-1 during Notch mediated skeletal muscle regeneration^30^. Furthermore, Numb inhibition of Notch signaling transduction had been implicated as a potential therapeutic target for prostate cancer^31^. Thus, the interaction between Numb and Notch have gained increasing interest, with Numb found to modulate Notch signaling by targeting Notch proteolytic degradation and its intracellular domains^32^. Additionally, in mouse hepatocytes, the Notch receptor and ligand are highly expressed during biliary regeneration^33^. In our previous study, inhibiting Notch signaling was found to inhibit CLF progression, with Numb expression found to be significantly reduced in a CLF model induced by BDL^12^. However, at present, no studies have confirmed the role of Numb in CLF. Thus, further characterizing the role of Numb in CLF could facilitate the development of further treatments. Herein, BMSC^*Numb*-KD^ transplantation was shown to promote CLF development (detailed data will be presented in another article).

BMSCs are exogenous hepatic stem cells and easily acquired^34–37^ and have a high degree of plasticity. Under specific conditions in vitro, BMSCs can be induced to differentiate into various functional cells, such as bone cells, hepatocytes and fat cells^38^. Furthermore, BMSCs have a low rejection level, rapid expansion in vitro, easy differentiation induction, and can easily have exogenous genes introduced^39^. In this study, *Numb* knockdown was performed in BMSCs by using RNAi, with these cells then transplanted into CLF rats induced by BDL. The results showed that BMSC^*Numb*-KD^ transplantation significantly increased the Hyp content and promotes α-SMA, Col I, TGF-β1, TNF-α, CK7 and CK19 expression in vivo. These findings indicate that BMSC^*Numb*-KD^ transplantation can promote CLF progression. Additionally, in the BMSC^NC^ group, EGFP was not co-localized with CK7 or CK19, but in BMSC^*Numb*-KD^ group, they were extensively co-localized in proliferating BECs, suggesting that *Numb* knockdown promotes the differentiation of BMSCs into BECs and thus promotes CLF progression.

### BMSC^***Numb***-KD^ transplantation reduces the anti-CLF effect of HQD

In our previous studies, HQD was found to inhibit CLF by inhibiting HPCs differentiation into BECs, and HQD was found to promote Numb expression in vivo and in vitro^14^. Therefore, *Numb* gene of hepatic stem cells might be related to HQD against CLF effect. To prove this hypothesis, HQD was administered after BMSC^*Numb*-KD^ transplantation into rats with CLF. In the BMSC^*Numb*-KD^ group with HQD treatment, although liver fibrosis has improved compared with the BMSC^*Numb*-KD^ group, Hyp content and the mRNA levels of α-SMA, TGF-β1, TNF-α, ColI,CK7 and CK19 were significantly increased compared to that in the HQD group, suggesting that BMSC^*Numb*-KD^ transplantation into the liver promotes CLF by reducing Numb expression, thereby weakening the effect of HQD against CLF. However, in this case, the liver still had Numb expression as a result of not directly knocking out the *Numb* gene, thus the above experimental results were obtained.

To further examine the interaction between Numb of hepatic stem cell and HQD, EGFP (mark BMSC^NC-KD^ or BMSC^*Numb*-KD^) co-expression with CK19 or CK7 was examined. In the BMSC^*Numb*-KD^ group, EGFP co-stained with CK19, while this phenomenon was not obviously improved after HQD treatment. The same result was seen for EGFP/CK7, suggesting that HQD has no obvious intervention effect in BMSC^*Numb*-KD^ differentiation into BECs. This result was further substantiated using WB-F344 cells in vitro, with *Numb* knockdown enhancing CK19 expression and HQD treatment having no significant effect. As confirmed in vivo, these observations further indicated that hepatic stem cell with *Numb* knockdown reduces the effect of HQD on CLF progression.

In a previous study to examine human embryonic stem cells, Numb was found to be upregulated following Sal B treatment, thus further negatively regulating the Notch pathway and subsequently inhibiting biliary differentiation^40^. Considering this relationship between Numb and Notch signaling, changes in the Notch signaling resulting from Numb modulation were further examined. In our previous study, HQD was found to inhibit Notch signaling pathway activation^14^. Herein, only Notch-2 and DLL-4 mRNA levels were significantly lower in the HQD plus BMSC^*Numb*-KD^ group compared to that in BMSC^*Numb*-KD^ group, suggesting that BMSC^*Numb*-KD^ transplantation reduces the ability of HQD to inhibit Notch signal pathway activation.

When the Notch receptor binds to its ligand, γ-secretase cleaves the TNF-α, thereby converting enzyme and releasing NICD^41^, and subsequent translocation of the NICD to the nucleus to modulate downstream gene expression^42^. In this work, the changes in RBP-Jκ and Hes1 expression were examined. The results showed that in the HQD plus BMSC^*Numb*-KD^ group, Numb expression was reduced, while RBP-Jκ and Hes1 expressions were enhanced compared to that in HQD group in vivo. Furthermore, in vitro study showed that in the *Numb*-KD plus HQD group, Numb expression was increased, while RBP-Jκ and Hes1 expression were reduced compared to that in *Numb*-KD group. However, when compared to that in the HQD group, Numb expression was significantly reduced, while RBP-Jκ and Hes1 expressions were significantly enhanced. Based on these observations, HQD might inhibit Notch signaling pathway by promoting Numb expression and subsequently inhibiting bile duct hyperplasia and improving CLF.

On the other hand, E3 ubiquitin ligase plays an important role in Notch receptor regulation. LNX, as a Numb PTB-binding molecule, it was found to act as a RING finger-type E3 ubiquitin ligase, causes proteasome-dependent degradation of Numb and can enhance Notch signalling^25,43^. In contrast, ITCH, an E3 ubiquitin ligase that belongs to the HECT family, negatively regulates Notch signaling and promotes ubiquitination-dependent proteasomal degradation of the NICD. Furthermore, Numb can interact with ITCH to cooperatively enhance Notch ubiquitination and degradation, circumventing its nuclear localization and downstream activation of Notch1 target genes^26,44–46^. Therefore, we also observed the mRNA expression of LNX and ITCH. The results indicated that HQD could significantly inhibit the expression of LNX1 and increase the expression of ITCH. However, when BMSC cells were transplanted after *Numb* knockdown, the above positive effects of HQD disappeared, which further proved that Numb might be the key target of HQD against CLF.

In summary, in BDL-induced liver fibrosis, the intervention effect of HQD and BMSC was similar to DAPT, but the transplantation of BMSCs with *Numb* knockdown can significantly reduce the anti-CLF effects of HQD. This may be attributed to the weakening inhibitory effect of HQD on Notch signal pathway after BMSCs *Numb* knockdown. Therefore, HQD may inhibit the progress of CLF through the hepatic stem cell *Numb* gene and which will provide a new therapeutic approach for CLF. To be sure, one potential short coming of utilizing RNAi technology is that it is impossible to completely knock out *Numb*. Therefore, future studies should be focused on knockout the *Numb* gene and then examining the effect of HQD against CLF. On the other hand, both HQD and BMSC do have rather good effect against CLF, there will be better therapeutic effect if they are used in combination and it is our top issue in the follow-up study.

## Supplementary information

Supplementary Information.

## Data Availability

All data generated or analyzed during this study are included in this published article and its supplementary information files.
