# Supplementary material for: Hepatic stem cell Numb gene is a potential target of Huang Qi Decoction against cholestatic liver fibrosis
Source: Sci Rep. 2020 Oct 15;10:17486. doi: 10.1038/s41598-020-74324-1 (PMC7566460; doi:10.1038/s41598-020-74324-1)

**Hepatic stem cell *Numb* gene is a** **potential target of Huang Qi Decoction** **against cholestatic liver fibrosis**

Wen Xu^1,2#^, Yan-nan Xu^1,2#^, Xu Zhang^1,2^^#^, Ying Xu^1,2^, Xun Jian^1,2^, Jia-mei Chen^1,2^, Gao-feng Chen^1,2^, Hua Zhang^1,2^, Ping Liu^1,2,3§^, and Yong-ping Mu^1,2§^

1. Shuguang Hospital affiliated to Shanghai University of Traditional Chinese Medicine (TCM); Institute of Liver Diseases, Key Laboratory of Liver and Kidney Diseases, Shanghai University of TCM, Shanghai, China.

2. Shanghai key laboratory of TCM, Shanghai, China.

3. E-institute of Shanghai Municipal Education Commission, Shanghai University of TCM, Shanghai, China.

**^#^ Co-first author:** Wen Xu, Yan-nan Xu and Xu Zhang contributed equally to this study and share first authorship.

**^§^Correspondence to:** Prof. Yong-Ping Mu and Prof. Ping Liu, Shuguang Hospital Affiliated to Shanghai University of Traditional Chinese Medicine (TCM); Institute of Liver Diseases, Key Laboratory of Liver and Kidney Diseases, Shanghai University of TCM; Shanghai Key Laboratory of TCM. 528, Zhangheng Road, Pudong district, Shanghai 201203, P.R. China. Tel: (+86) 21-2025-6526, Fax: (+86) 21-2025-6521, E-mail: ypmu8888@126.com (Yong-ping Mu); Tel: (+86) 21-2025-6526, Fax: (+86) 21-2025-6521, E-mail: Liuliver@vip.sina.com (Ping Liu)

**Supplementary Materials**

**1. The fingerprint chromatogramsof HQD by UHPLC-Q-Orbitrap HRMS**

The chemical analysis of extract of HQD was analyzed by using ultra-high-performance liquid chromatography-Q exactive hybrid quadrupoleorbitrap high-resolution accurate mass spectrometric (UHPLC-Q-Orbitrap HRMS, Thermo Fisher Scientific Inc., Grand Island, NY, USA). The UHPLC was Thermo Scientific Dionex Ultimate 3000 and controlled by Chromeleon 7.2 Software. The cooling autosampler was set at 10°C and protected from light, and the column heater was set at 40°C. A Waters ACQUITY UPLC HSS T3 column (2.1 × 100 mm, 1.8 μm) was employed and the column temperature was set at 40°C. The mobile phase consisted of A (acetonitrile) and B (0.1% formic acid) at a flow rate of 0.4 mL·min^−1^ and eluted with gradient elution: 0-2 min (2% A), 2-11 min (2%-95% A), 11-13min (95% A), 13-14 min (2% A). The injection volume was 5 μL.

The mass spectrometer Q-Orbitrap system was connected to the UHPLC system via a heated electrospray ionization and controlled by Xcalibur 4.1 software that was used for data capture and analysis. The electrospray ionization source was operated in negative and positive ionization mode. The optimized parameters of mass spectrometry were: capillary temperature: 325°C; sheath gas (N_2_) flow rate: 45 arbitrary units; auxiliary gas (N_2_) flow rate: 8 arbitrary units; sweep gas flow rate: 0 arbitrary units; spray voltage: 2.8 kV (negative) and 3.5 kV (positive); S-lens RF level: 50V; auxilliary gas heater temperature, 300°C; scan mode: full MS: scan range: 100-1500 *m/z*. The typical chromatographic fingerprints of extract of Huangqi Decotion were showed in Supplementary Fig. 1, and four characteristic compounds calycosin-7-glucoside (Rt 6.87min, [M + HCOOH-H]^-^*m/z* 491.11840, [M + H]^+^*m/z* 447.12857, characteristic component of *Astragalusmongholicus*Bunge.), calycosin (Rt 8.59 min, [M-H]^-^*m/z* 283.06009, [M + H]^+^*m/z* 285.07575, characteristic component of *Astragalusmongholicus*Bunge.), astragaloside IV (Rt 9.76min, [M+HCOOH-H]^-^*m/z* 829.45801, [M + H]^+^*m/z* 785.46818, characteristic component of *Astragalusmongholicus*Bunge.) and glycyrrhizic acid (Rt 9.89min, [M-H]^-^*m/z* 821.39541, [M + H]^+^*m/z* 823.41106, characteristic component of *Glycyrrhizauralensis* Fisch) were identified.


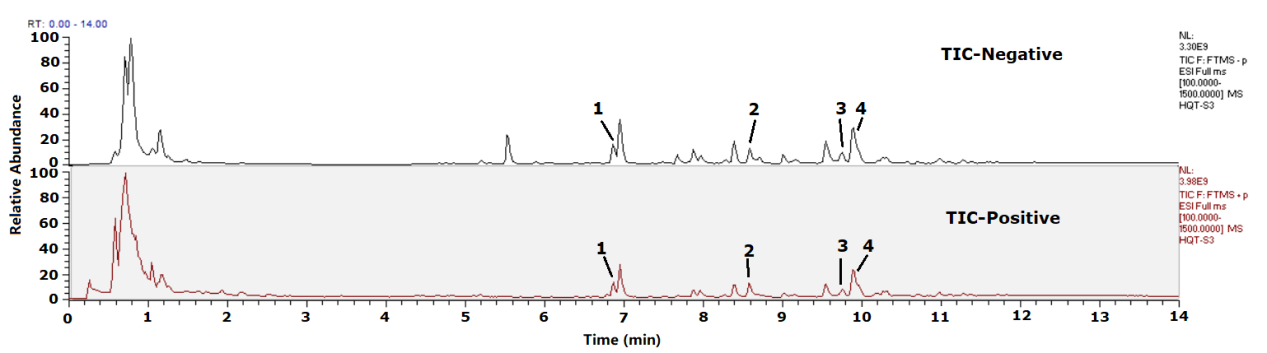


**Supplementary Fig. 1. The fingerprint chromatograms of extract of HQD in negative and positive model by UHPLC-Q-Orbitrap HRMS**: 1, calycosin-7-glucoside; 2, calycosin; 3, astragaloside IV; 4, glycyrrhizic acid.

**2. Original western blot images**


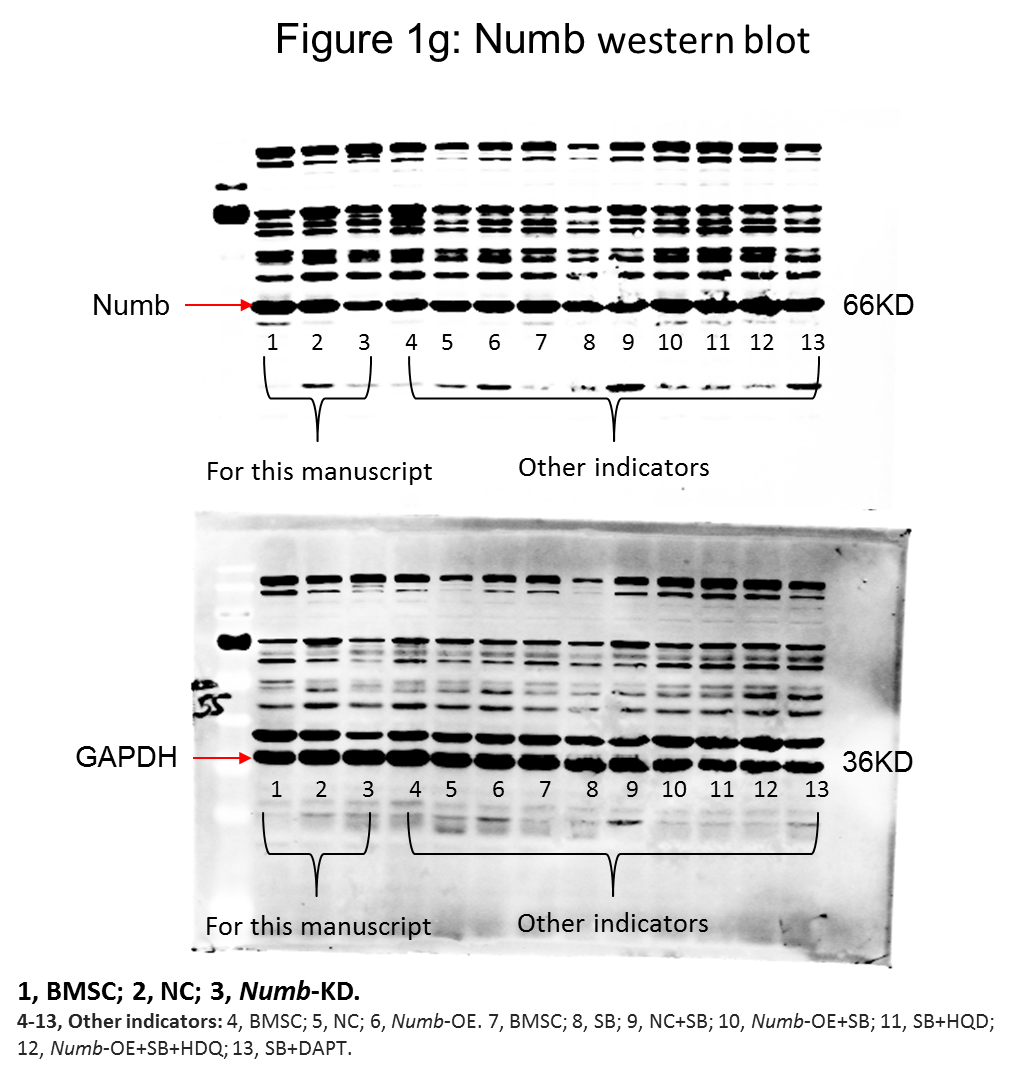


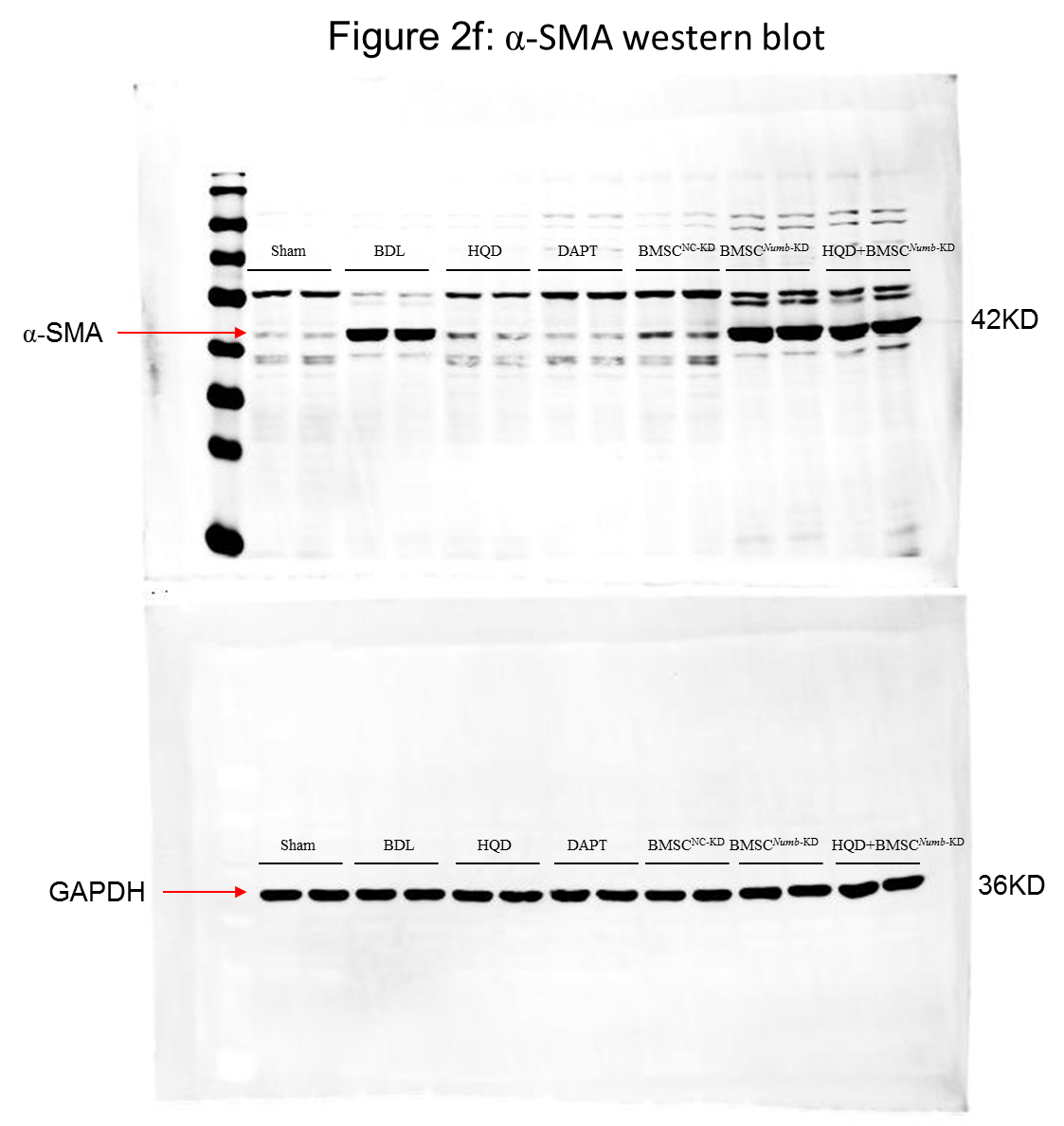


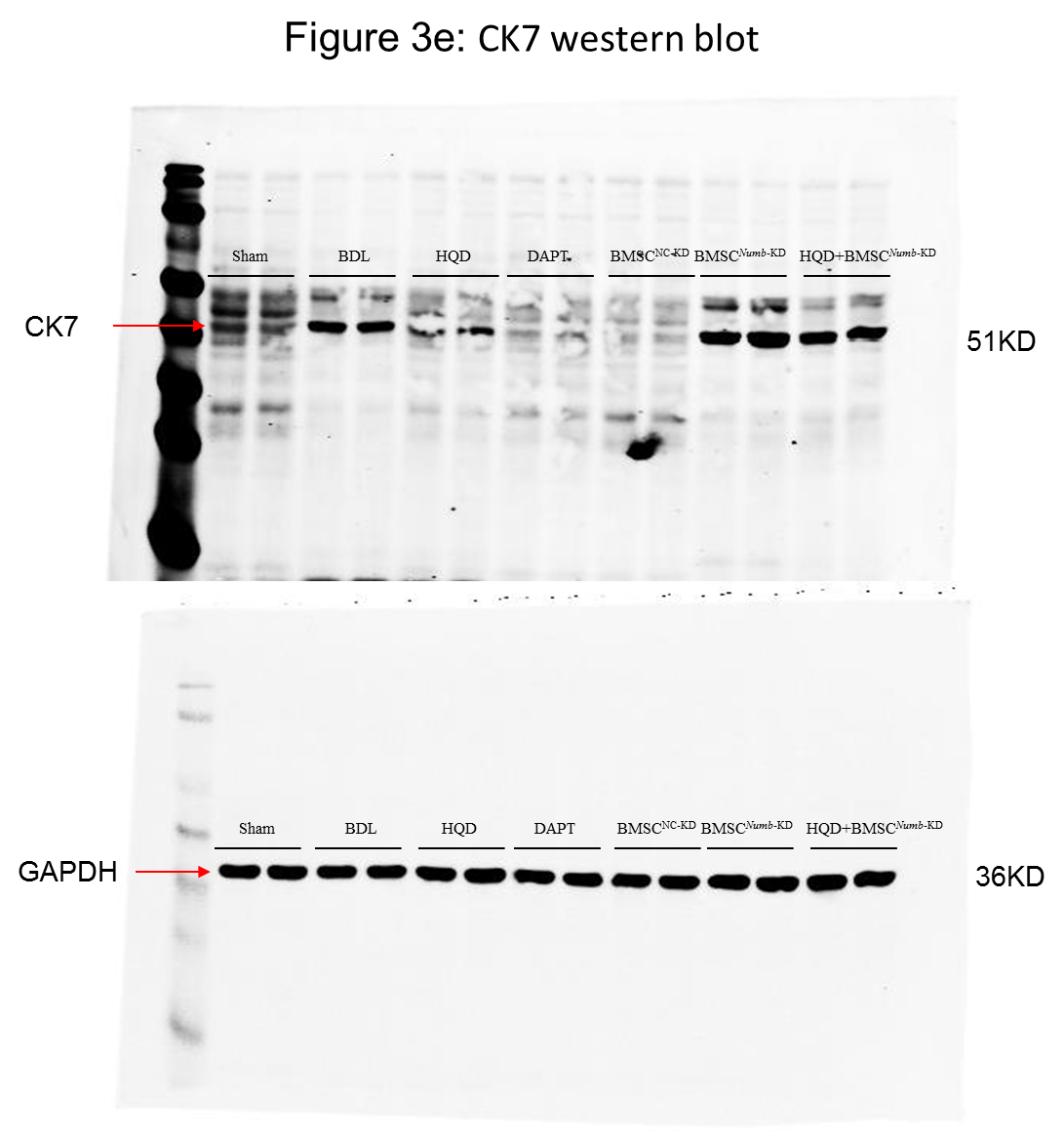


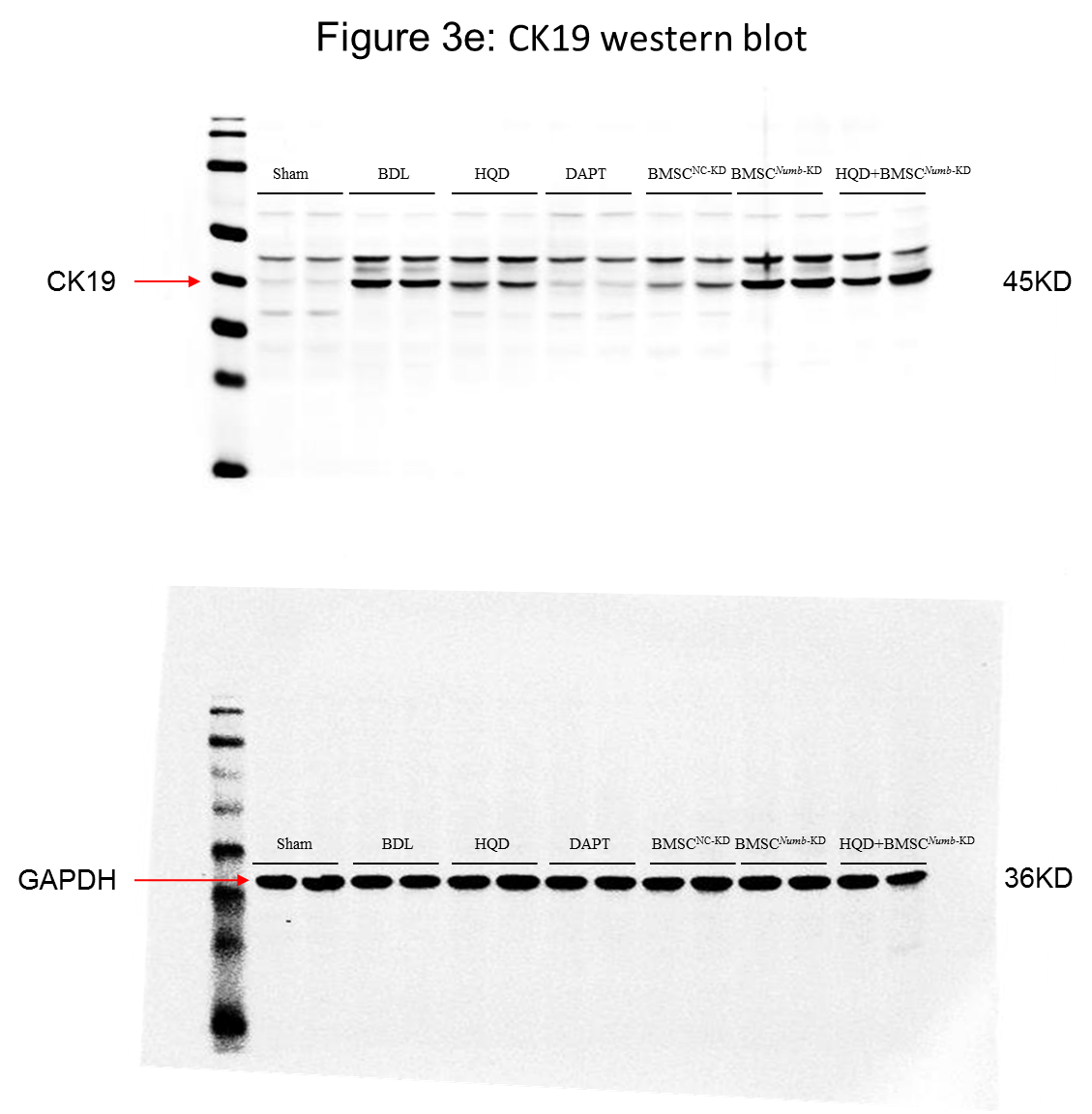


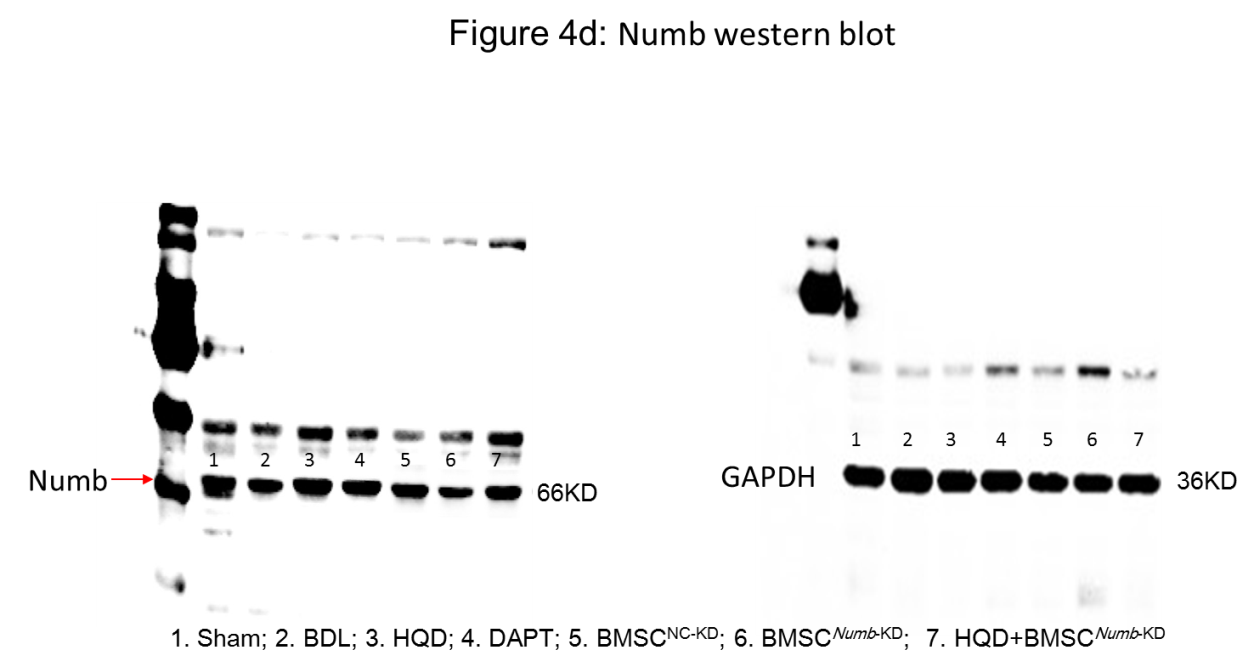


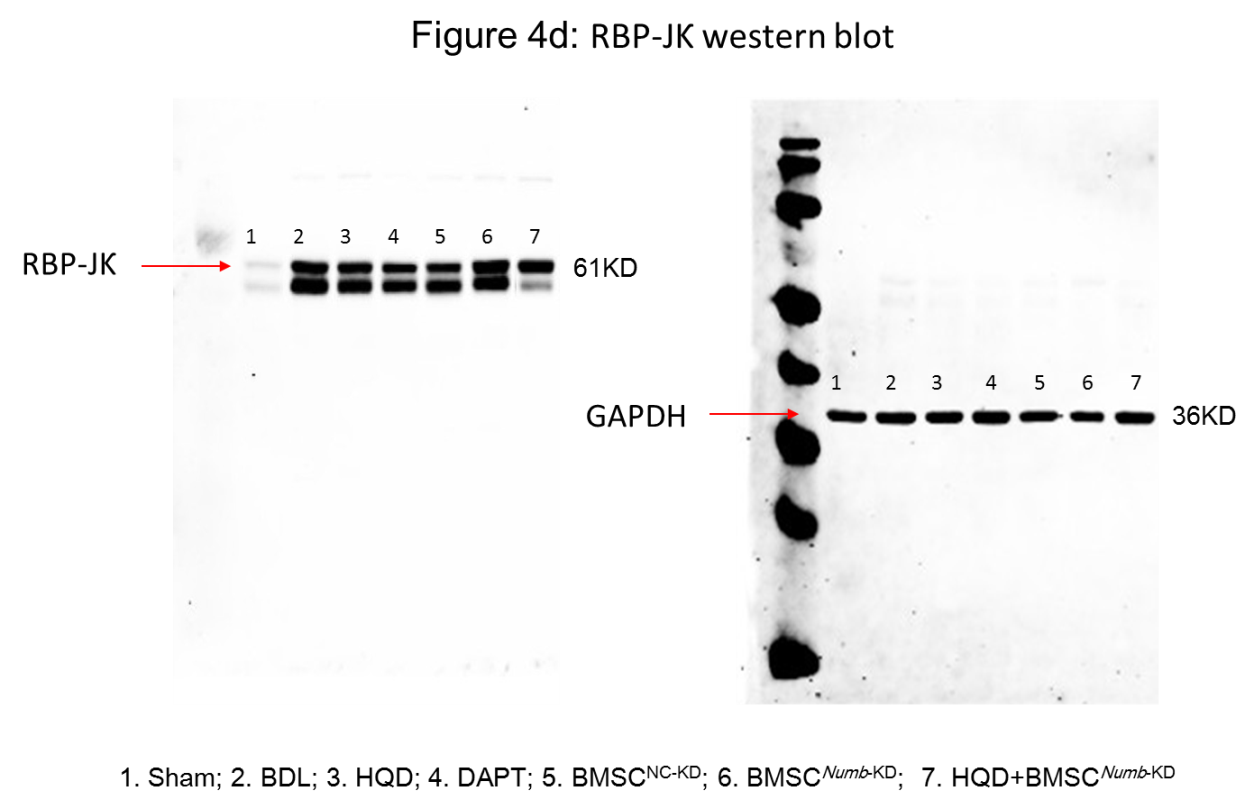


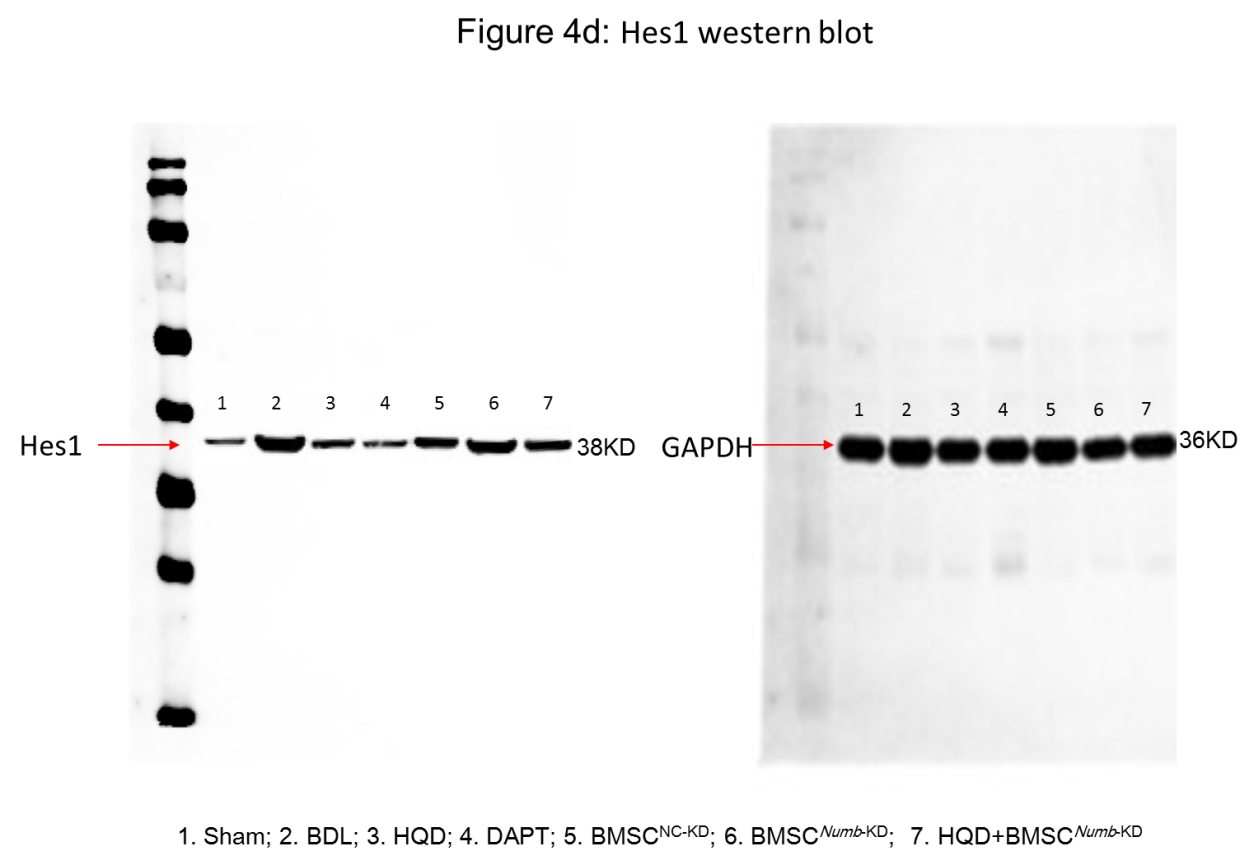


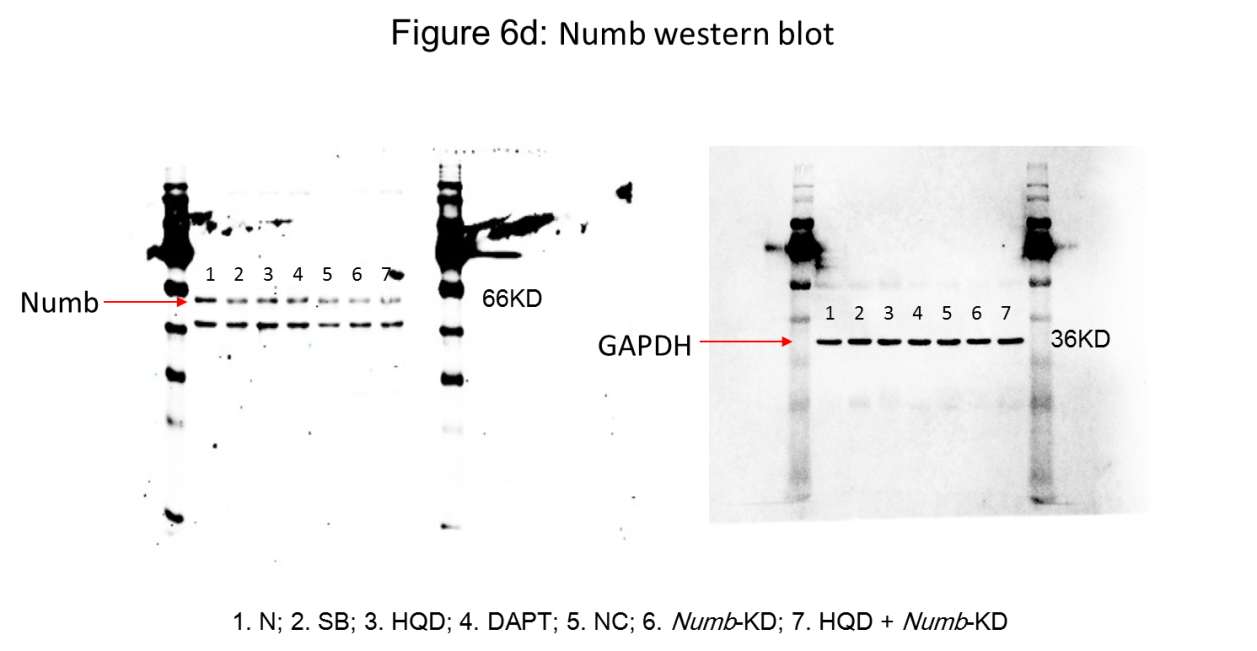


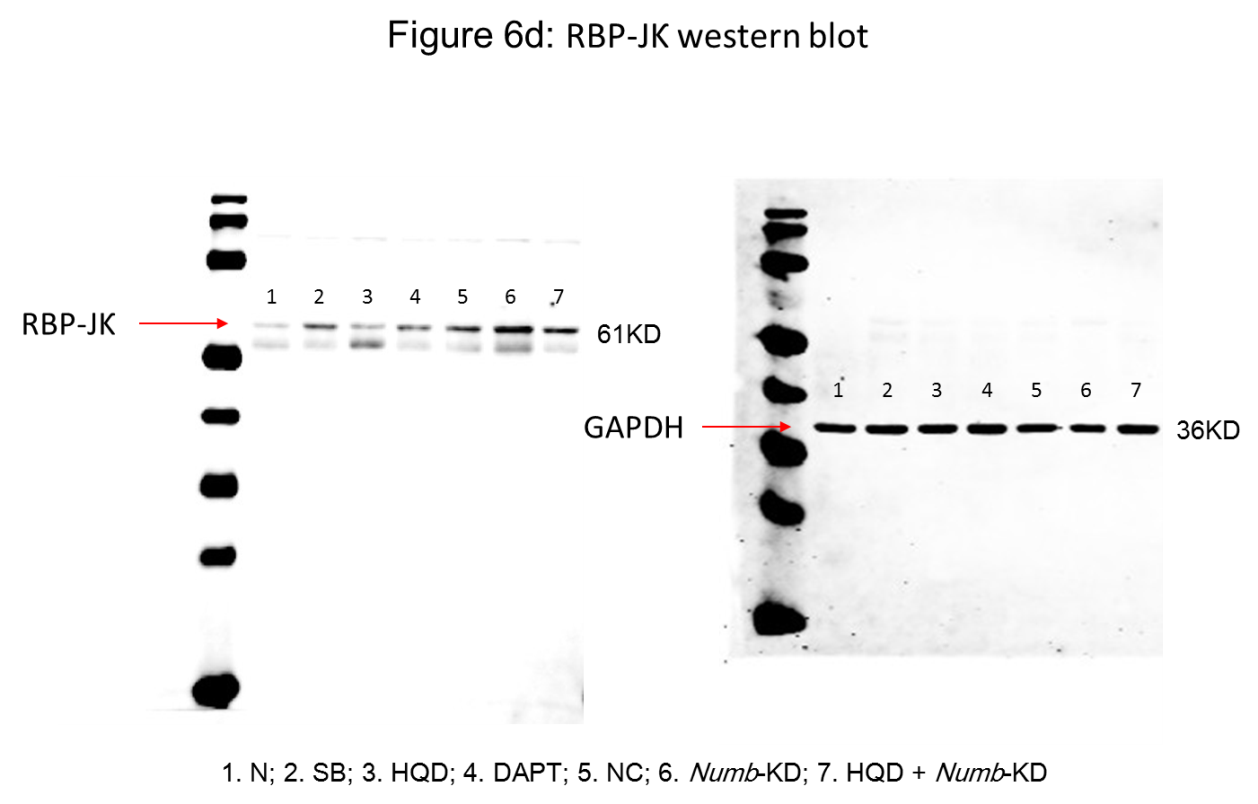


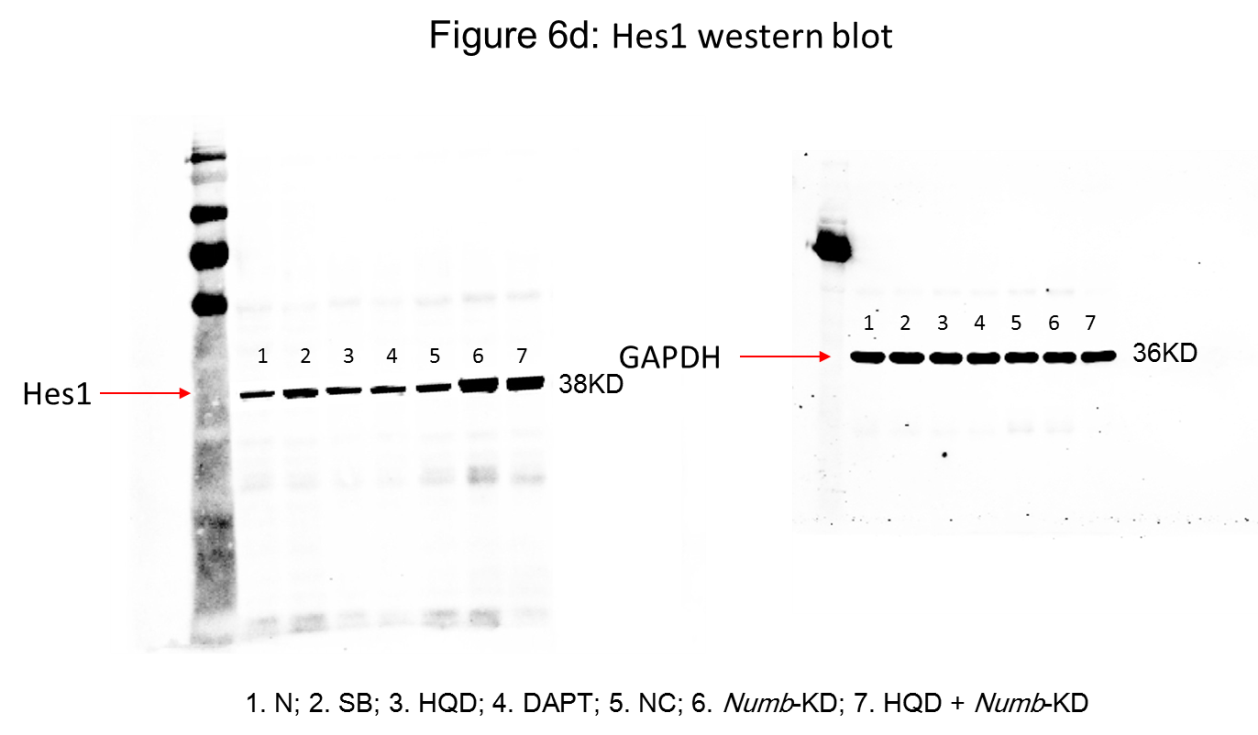

Supplement: Supplementary file 1 — Supplementary Information. [file 41598_2020_74324_MOESM1_ESM.docx]
